# CD73 inhibition overcomes adaptive immune resistance to PARP inhibition in models of prostate cancer

**DOI:** 10.1172/JCI203785

**Published:** 2026-08-18

**Authors:** Ping Xie, Renqiang Ma, Minghui Zhang, Jie Fan, Hui Tang, Longzhen Song, Yong Wan, Timothy M. Kuzel, Deyu Fang, Weiguo Cui, Jennifer D. Wu, Sarki A. Abdulkadir, Yi Zhang, Akash Patnaik, Bin Zhang

**Affiliations:** 1 Department of Medicine, Hematology/Oncology Division, Robert H. Lurie Comprehensive Cancer Center, Northwestern University Feinberg School of Medicine, Chicago, Illinois, USA.; 2 Otorhinolaryngology Hospital, The First Affiliated Hospital of Sun Yat-Sen University, Guangzhou, China.; 3 Department of Obstetrics and Gynecology, The First Affiliated Hospital of Zhengzhou University, Zhengzhou, China.; 4 Department of Pharmacology and Chemical Biology, Emory University School of Medicine, Atlanta, Georgia, USA.; 5 Department of Pathology and; 6 Department of Urology, Northwestern University Feinberg School of Medicine, Chicago, Illinois, USA.; 7 Biotherapy Center, The First Affiliated Hospital of Zhengzhou University, Zhengzhou, China.; 8 Section of Hematology/Oncology, Department of Medicine, University of Chicago, Chicago, Illinois, USA.

**Keywords:** Immunology, Oncology, Adaptive immunity, Cancer immunotherapy

## Abstract

Metastatic castration-resistant prostate cancer (mCRPC) remains a leading cause of cancer-related mortality in men. Although poly(ADP-ribose) polymerase (PARP) inhibitors are approved for mCRPC patients with homologous recombination repair (HRR) deficiencies, clinical trials combining olaparib with PD-1/PD-L1 inhibitors showed limited efficacy in unselected populations. To investigate the immunomodulatory effects of PARP inhibitors in an unbiased manner, we performed bulk RNA-seq on HRR-proficient MycCaP cells treated with the PARP inhibitor olaparib versus vehicle control. Transcriptomic analysis revealed robust upregulation of CD73 (*NT5E*), an ectoenzyme and emerging immune checkpoint that generates extracellular adenosine, suggesting an adaptive mechanism that undermines olaparib efficacy and promotes immunosuppression. CD73 induction by olaparib was validated in human and mouse prostate cancer cell lines, with more pronounced effects in HRR-compromised PTEN-KO cells. Mechanistically, olaparib-driven CD73 expression was mediated through DNA damage–activated ATR/CHEK1/IRF1 and TGF-β1/AKT signaling pathways. In parallel, olaparib enhanced tumor immunogenicity by activating type I IFN signaling and antigen presentation machinery. In vivo, combining olaparib with CD73 blockade significantly delayed tumor growth, improved T cell infiltration, and augmented CD8^+^ T cell effector function across HRR-proficient and PTEN-KO prostate cancer models. These findings identify olaparib-induced CD73 upregulation as an adaptive resistance mechanism and support olaparib plus CD73 blockade as a promising therapeutic strategy for advanced prostate cancer, irrespective of HRR status.

## Abstract 2

<style type=”text/css”>a { text-decoration: none; color: #464feb; } tr th, tr td { border: 1px solid #e6e6e6; } tr th { background-color: #f5f5f5; } </style> <div>A prostate cancer drug triggers a defense that helps tumors evade immune attack. Blocking this defense improves immune responses and slows tumor growth.</div>

## Graphical abstract

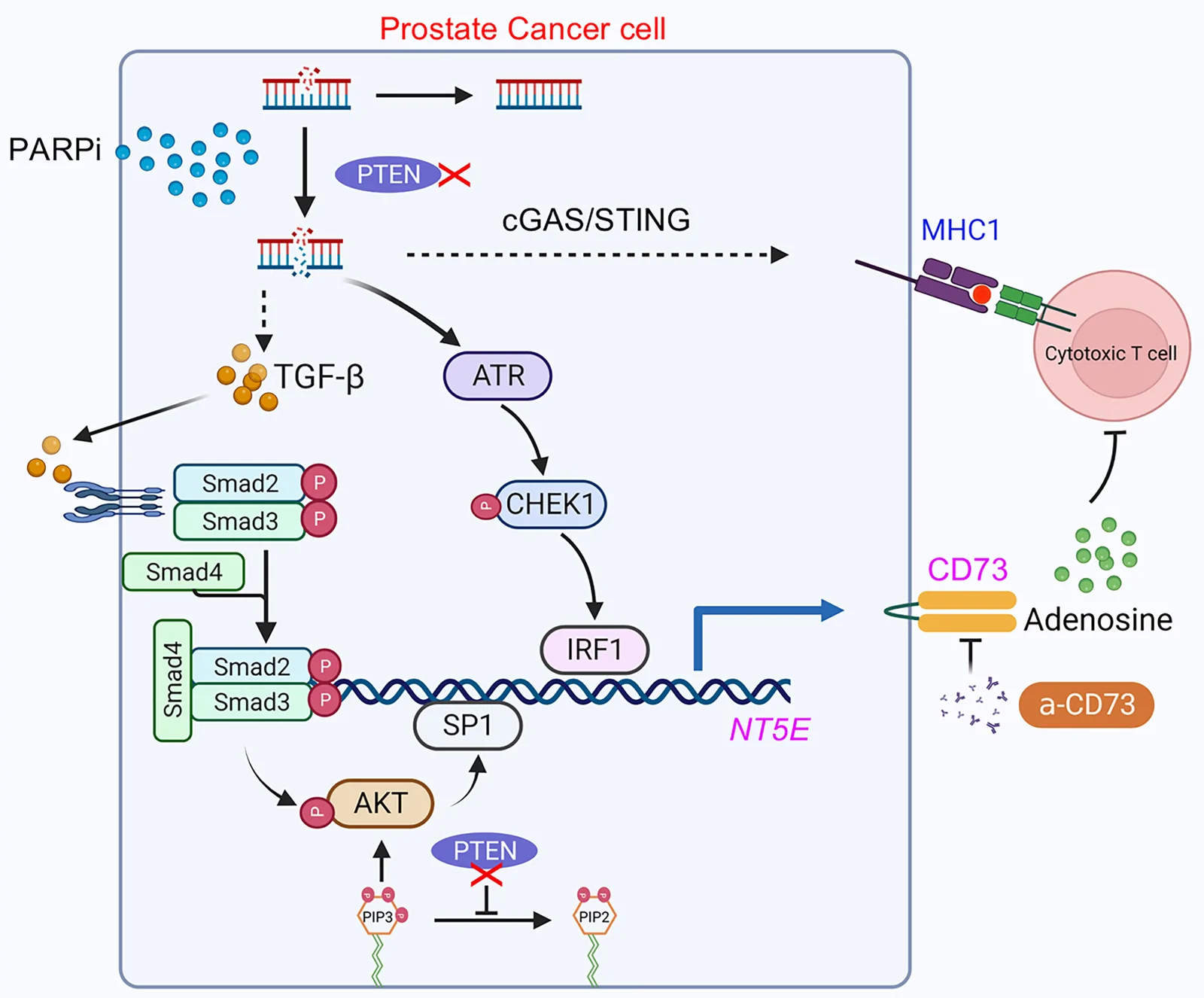

## Introduction

Poly(ADP-ribose) polymerase (PARP) inhibition (PARPi) has emerged as an effective therapeutic strategy for tumors with genomic instability in dsDNA repair genes through the induction of synthetic lethality (1, 2). Several PARP inhibitors — including olaparib, niraparib, talazoparib, rucaparib, and veliparib — are under clinical development for malignancies harboring homologous recombination repair (HRR) gene mutations, such as *BRCA1/2* (3). Among these, olaparib is the most extensively studied in metastatic castration-resistant prostate cancer (mCRPC) (4, 5). Supported by the landmark phase III PROfound trial (6), olaparib has recently received US FDA approval for the treatment of HRR-mutated mCRPC. Beyond monotherapy, several combinations of PARP inhibitors with androgen receptor pathway inhibitors have now also been approved. Despite these advances, the preclinical and clinical investigation of olaparib remains incomplete (7, 8). In particular, whether PARPi exerts therapeutic effects in advanced prostate cancer independent of DNA repair deficiency — and the immunoregulatory mechanisms contributing to PARPi resistance — remain poorly understood (9). Thus, identifying optimal combination strategies to enhance PARPi sensitivity and overcome resistance is an urgent priority.

Emerging evidence suggests a link between DNA damage repair and immune regulation. For example, PARPi-mediated targeting of DNA repair proteins has been shown to increase PD-L1 expression via GSK3β inactivation (10, 11) and activation of the ATM/ATR/CHK1 pathway (12, 13). *BRCA1/2* defects have also been associated with elevated PD-L1 expression in ovarian cancer (14, 15). Preclinical studies have demonstrated synergy between PARPi and immune checkpoint inhibitors (ICIs) in breast and lung cancer models (13), providing a rationale for clinical trials combining PARPi with ICI. However, in mCRPC, trials of pembrolizumab plus olaparib (16) and nivolumab plus rucaparib (17) have yielded limited benefit in unselected populations. These disappointing outcomes suggest the existence of compensatory immunosuppressive mechanisms triggered by PARPi that may counteract potential immunostimulatory effects. Defining these adaptive responses is essential for rational design of next-generation PARPi-based immunotherapies.

To uncover immunomodulatory mechanisms induced by PARPi in HRR-proficient prostate cancer, we performed unbiased transcriptomic profiling of MycCaP cells (18) treated with olaparib. This analysis revealed robust upregulation of CD73 (*NT5E*), an ecto-5′-nucleotidase that catalyzes extracellular adenosine production — a potent suppressor of antitumor immunity (19, 20). CD73-mediated adenosinergic signaling promotes tumor growth and metastasis (21–24) and has been associated with aggressive phenotypes in prostate cancer (25). Functionally, CD73 acts as an immune checkpoint, inhibiting effector T cell and NK cell activity, impairing DC maturation, and fostering immunosuppressive myeloid populations across multiple solid tumors, including prostate cancer (26, 27). Thus, PARPi-driven CD73 induction represents a previously unrecognized adaptive resistance mechanism that may blunt antitumor immunity and limit the efficacy of PARPi-based immunotherapy combinations.

We further observed that PARPi-induced CD73 upregulation occurs broadly across mouse and human prostate cancer cell lines, including HRR-proficient tumors and PTEN-KO cells, an aggressive subtype associated with immune evasion and poor outcomes (28, 29). Combining olaparib with anti-CD73 antibody therapy significantly enhanced antitumor immune activity and delayed tumor progression in both HRR-proficient and PTEN-KO tumor models. These findings identify CD73 induction as a targetable adaptive response to olaparib and support the development of olaparib plus CD73 blockade as a promising therapeutic strategy for prostate cancer across diverse molecular backgrounds.

## Results

### Olaparib induces CD73 upregulation in prostate cancer cells, conferring adaptive immune resistance.

To investigate the molecular mechanisms underlying the immunomodulatory effects of olaparib in prostate cancer, we performed bulk RNA-seq on MycCaP cells treated with olaparib (20 μM, 24 h). Gene pathway enrichment analysis revealed a multifaceted landscape of olaparib-mediated immune-dependent and -independent activities in treated tumor cells (Figure 1A). Notably, olaparib treatment differentially affected the transcriptional expression of a panel of immunoinhibitory and immunostimulatory molecules (Figure 1B). Among these, *Nt5e* (encoding CD73) emerged as the most significantly upregulated gene following olaparib treatment (Figure 1B).

RT-qPCR analysis confirmed a robust increase in *Nt5e* mRNA expression following olaparib treatment in MycCaP cells (Figure 1C). Several reports have indicated that reduced levels or deletion of PTEN are associated with decreased homologous recombination efficiency (30–32), supporting the rationale for treating PTEN-deficient prostate cancer with agents targeting homologous recombination defects such as olaparib. However, conflicting evidence (33, 34) has shown that PTEN loss has no effect on PARPi sensitivity, particularly in advanced prostate cancer (35) either with or without ICI in both preclinical and clinical studies (36). Interestingly, the basal expression levels of *Nt5e* were higher in Myc-CaP-PTEN–KO cells than parental cells, and *Nt5e* levels were further upregulated following olaparib treatment (Figure 1C). A similar upregulation of *Nt5e* was observed in DKO (*Trp53/Rb1* double knockout) prostate tumor cells (Figure 1C) (37).

Flow cytometry analysis confirmed a dose-dependent increase in surface CD73 levels in MycCaP, MycCaP-PTEN–KO, and DKO cells following olaparib treatment (Figure 1D). Consistently, Western blot analysis demonstrated a dose-dependent upregulation of CD73 protein in MycCaP and MycCaP-PTEN–KO cells (Figure 1E). A similar induction of surface CD73 by olaparib was also observed in the human prostate cancer cell lines DU145 and PC3 (Supplemental Figure 1A; supplemental material available online with this article; https://doi.org/10.1172/JCI203785DS1). In addition, talazoparib — another clinically relevant PARP inhibitor — likewise increased CD73 expression in both MycCaP and MycCaP-PTEN–KO cells (Supplemental Figure 1B).

To determine the role of PARP in regulating CD73, we generated *Parp1*-knockdown (*Parp1*-KD) MycCaP cells, given the predominant expression of *Parp1* (Supplemental Figure 1C). Genetic ablation of *Parp1* alone was sufficient to induce CD73 expression, effectively mimicking pharmacological inhibition. Notably, subsequent treatment with olaparib did not further increase CD73 expression in the *Parp1*-KD cells compared with treated nontarget controls (Supplemental Figure 1D), indicating that the olaparib-mediated upregulation of CD73 is primarily driven by PARP1 inhibition. Consistently, a significant negative correlation between *PARP1* and *NT5E* gene expression was observed across human prostate cancer cell lines and primary tumor samples (Supplemental Figure 1E).

Bulk RNA-seq GSEA revealed that olaparib treatment markedly enriched pathways associated with antigen processing and presentation (APP) and type I IFN responses in MycCaP cells (Figure 1F and Supplemental Figure 2, A and B). Consistent with these transcriptomic changes, flow cytometry showed that olaparib induced a dose-dependent increase in surface MHC-I (H-2Kq) expression in both MycCaP and MycCaP-PTEN–KO cells (Figure 1G).

To examine the immunoregulatory role of CD73 induced by olaparib, olaparib-treated MycCaP cells with or without CD73 KD (Supplemental Figure 2C) were cocultured with MycCaP tumor-reactive CD8^+^ T cells, as outlined in Figure 1H. Either olaparib treatment or CD73 KD increased IFN-γ and TNF-α production and upregulated CD25 expression in cocultured tumor-reactive CD8^+^ T cells, while CD73 KD further amplified the olaparib-mediated immunostimulatory effects (Figure 1I). Likewise, tumor-reactive CD8^+^ T cell–mediated cytotoxicity against MycCaP tumor cells was enhanced by either olaparib pretreatment or CD73 KD, and CD73 KD further augmented olaparib-mediated T cell killing (Figure 1J). Together, these data suggest that adaptive upregulation of tumor CD73 induced by olaparib hinders tumor cell recognition and killing by tumor-reactive CD8^+^ T cells.

### Olaparib induces tumor CD73 expression through the ATR/IRF1 signaling pathway.

As CD73 contributes to intracellular NAD^+^ pools — an essential cofactor for PARP enzymatic activity required for DNA repair and genomic stability (38) — we next examined how PARPi affected DNA damage and CD73 regulation in prostate cancer cells. Flow cytometry analysis revealed a marked increase in the DNA double-strand break marker p-H2AX following olaparib treatment in MycCaP cells (Figure 2A). Immunofluorescence staining further demonstrated strong spatial colocalization of p-H2AX and CD73 signals, indicating that CD73 upregulation preferentially occurs in DNA-damaged tumor cells (Figure 2B). Flow cytometry confirmed a positive correlation between surface CD73 expression and intracellular p-H2AX levels (Figure 2C). The presence of CD73 induction in γH2AX^–^ cells suggests that CD73 upregulation likely reflects a combination of cell-intrinsic DNA damage signaling and non–cell-autonomous or stress-mediated effects, rather than being exclusively restricted to γH2AX^+^ cells.

To delineate the DNA damage–associated signaling pathways induced by PARPi, we performed GSEA on bulk RNA-seq data. Olaparib treatment enriched gene sets associated with HRR and ATR activation (Figure 2D and Supplemental Figure 3, A and B), suggesting a potential role for ATR in downstream transcriptional regulation of CD73 expression. Consistent with this notion, ATR inhibition partially attenuated olaparib-induced CD73 in MycCaP and, to a lesser extent, in MycCaP-PTEN–KO cells, whereas ATM inhibition had no detectable effect (Figure 2E). ATR inhibition comparably reduced CD73 expression under both baseline (control) and olaparib-treated conditions (Figure 2E), indicating a role for ATR signaling in maintaining constitutive CD73 expression. However, its additional role in PARPi-induced CD73 upregulation cannot be excluded.

Given that IRF1 is a downstream effector of ATR signaling (39) and is predicted to bind the *Nt5e* (CD73) promoter (Supplemental Figure 3C), we next assessed its involvement in CD73 induction. Olaparib treatment increased *Irf1* mRNA expression in both MycCaP and MycCaP-PTEN–KO cells in a dose-dependent manner (Figure 2F). ChIP assays showed significantly enhanced IRF1 occupancy at 2 predicted *Nt5e* promoter binding sites following olaparib treatment, and this increase was reversed by ATR inhibition (Figure 2G). To functionally validate IRF1’s role, we generated IRF1-KD prostate cancer cells. IRF1 KD significantly reduced olaparib-induced CD73 upregulation compared with nontargeting controls in both MycCaP and MycCaP-PTEN–KO cells, while having minimal effect on baseline CD73 levels in the absence of olaparib (Figure 2H). This suggests that IRF1 likely plays a more specific role in mediating PARPi-induced CD73 expression rather than constitutive expression. Importantly, in IRF1-KD cells, ATR inhibition did not further decrease CD73 expression under olaparib treatment (Figure 2I). This lack of additive effect indicates that ATR and IRF1 likely operate within the same linear signaling pathway. Together, these results support a model in which ATR/IRF1 signaling contributes specifically to olaparib-induced CD73 upregulation, while ATR may also independently sustain basal CD73 expression.

To assess whether ATR/IRF1 signaling operates independently of canonical cGAS/STING/type I IFN signaling, we pretreated tumor cells with the selective STING inhibitor STING-IN-2 (40) prior to olaparib treatment. STING inhibition markedly attenuated the induction of the canonical STING target MHC-I (Supplemental Figure 4A), confirming effective pathway blockade. In contrast, STING-IN-2 only slightly reduced olaparib-induced CD73 expression (Supplemental Figure 4B). Notably, ATR inhibition led to a more substantial decrease in CD73 levels (Figure 2E). These results suggest that while cGAS/STING/type I IFN signaling may contribute in part, CD73 upregulation is more strongly dependent on ATR signaling, supporting the existence of a partially distinct ATR/IRF1 regulatory axis.

Taken together, these findings reveal that olaparib induces DNA damage, activates ATR signaling, and promotes IRF1-dependent transcriptional upregulation of CD73 in prostate cancer cells. Nevertheless, additional mechanistic pathways are likely to contribute to CD73 induction by olaparib.

### Olaparib regulates CD73 expression through the TGF-β/AKT signaling axis.

Further transcriptomic analysis revealed elevated gene sets for components of the TGF-β pathway upon olaparib treatment (Figure 3A). Indeed, increased levels of TGF-β1 (Figure 3B) and its downstream effector pSMAD2 (Figure 3C) were observed in olaparib-treated MycCaP and MycCaP-PTEN–KO cells compared with controls. Flow cytometry analysis showed a positive correlation between CD73 and pSMAD2 expression (Supplemental Figure 4C), consistent with previous reports linking TGF-β signaling to CD73 regulation (41–43). Moreover, pharmacologic inhibition of the TGF-β receptor partially reduced olaparib-induced CD73 upregulation (Figure 3D), confirming a role for TGF-β signaling in mediating CD73 induction following olaparib.

Given the markedly elevated CD73 expression in PTEN-deficient MycCaP cells, and considering that PTEN loss is known to activate AKT signaling, we next assessed whether AKT contributes to CD73 regulation. Both Western blot analysis (Figure 3E) and flow cytometry (Supplemental Figure 4D) revealed a significant increase in AKT phosphorylation (pAKT) upon olaparib treatment, and CD73 expression positively correlated with pAKT levels (Supplemental Figure 4E). Pharmacologic inhibition of AKT significantly mitigated olaparib-induced CD73 expression in MycCaP cells (Figure 3F).

To determine whether TGF-β and AKT function within the same signaling axis, we evaluated the effect of inhibiting one pathway on activation of the other. TGF-β pathway inhibition abrogated the olaparib-induced increase in pAKT (Figure 3G), whereas AKT inhibition did not alter pSMAD2 levels in the absence or presence of olaparib (Supplemental Figure 4F). Furthermore, AKT inhibition did not affect the reduction in CD73 induced by TGF-β receptor blockade (Figure 3H). These results indicate that olaparib activates AKT signaling downstream of TGF-β, positioning AKT as an effector of TGF-β–mediated CD73 upregulation.

SP1, a key transcription factor downstream of AKT signaling (44, 45), is predicted to bind the Nt5e promoter (Supplemental Figure 3C). An earlier study reported that SP1 motifs are required for Nt5e gene upregulation (46). Thus, we speculated that olaparib-induced CD73 expression depends, at least in part, on AKT hyperactivation and subsequent SP1-mediated transcriptional activity. Supporting this hypothesis, ChIP assays showed SP1 binding at the −17 to −25 region of the *Nt5e* promoter, and olaparib treatment increased SP1 enrichment at this site (Figure 3I), whereas olaparib did not alter overall SP1 protein levels (Supplemental Figure 4G). Notably, inhibition of either TGF-β or AKT signaling abolished SP1 binding to the *Nt5e* promoter (Figure 3I), implicating a TGF-β/AKT/SP1 transcriptional axis in driving CD73 induction upon olaparib treatment. The induction of TGF-β by olaparib observed in tumor cell monocultures supports the notion that activation of the TGF-β/AKT/SP1 axis occurs in a tumor cell–autonomous manner. The transcriptional upregulation of TGF-β is possibly driven by PARPi-induced DNA damage–associated cellular senescence or a senescence-associated secretory phenotype (47, 48), which could promote TGF-β signaling to drive downstream AKT-SP1–mediated CD73 expression.

In addition, combined inhibition of TGF-β and ATR more effectively reversed olaparib-induced CD73 expression than inhibition of either pathway alone (Figure 3J), while ATR inhibition did not alter pSMAD2 levels (Supplemental Figure 4H). Consistent with these findings in murine tumor cells, treatment with an ATR inhibitor, a TGF-β inhibitor, or an AKT inhibitor each partially attenuated olaparib-induced CD73 upregulation in human CRPC DU145 cells (Supplemental Figure 5, A–C). Collectively, these results demonstrate that olaparib drives CD73 expression through 2 parallel, nonredundant mechanisms: the TGF-β/AKT/SP1 axis and the ATR/IRF1 pathway.

### Combination of olaparib and CD73 blockade therapy enhances antitumor immunity and suppresses tumor growth in a CD8^+^ T cell–dependent manner.

The observation that olaparib treatment upregulates CD73 in prostate cancer cells and counteracts CD8^+^ T cell antitumor activity (Figure 1, H–J) provides strong rationale for testing combined olaparib and CD73 blockade in vivo. Indeed, this combination more effectively inhibited tumor growth and improved survival compared with either monotherapy across multiple tumor models, including MycCaP, MycCaP-PTEN–KO, and DKO (Figure 4A). In contrast, neither anti–PD-1 monotherapy nor the combination of olaparib with anti–PD-1 affected tumor growth (Supplemental Figure 6A) or survival (Supplemental Figure 6B), consistent with emerging clinical data (16, 17).

To investigate the mechanistic basis of the cooperation between olaparib and CD73 blockade (Supplemental Figure 7A) and to characterize treatment-associated changes in antitumor immunity, we performed multiparameter spectral cytometry (CyTEK, Aurora) to analyze tumor-infiltrating T cells (both CD4^+^ and CD8^+^) and other key immune subsets (Figure 4B). While the overall frequencies of B cells, NK cells, tumor-associated macrophages (TAMs), DCs, and myeloid-derived suppressor cells (MDSCs) remained largely unchanged across treatment groups (Supplemental Figure 7, B–F), combination therapy significantly increased CD3^+^, CD4^+^, and CD8^+^ T cell infiltration (Figure 4C). This was accompanied by selective expansion of stem-like progenitor-exhausted CD8^+^ T cells (Tpex) and reduced expression of the exhaustion-associated transcription factor TOX (Figure 4D). DC activation was also enhanced, as evidenced by increased CD40 expression (Figure 4E). Additionally, PD-L1 expression on PMN-MDSCs was reduced following combination treatment (Figure 4F), whereas PD-L1 expression on monocytic MDSCs, TAMs, and DCs remained unchanged (Supplemental Figure 7, G–I).

Furthermore, CD73 KD sensitized MycCaP tumors to olaparib in vivo (Figure 4G), confirming a tumor-promoting role for CD73 during olaparib therapy. Immunohistochemistry analysis revealed that the combination of olaparib and CD73 KD significantly increased the infiltration of total CD8^+^ T cells and cytotoxic granzyme B^+^ CD8^+^ T cells (Figure 4, H–J), supporting enhanced CD8^+^ T cell–mediated antitumor immunity.

The stronger antitumor efficacy observed with CD73 KD (Figure 4G) compared with anti-CD73 antibody treatment (Figure 4A) likely reflects the difference between complete genetic deletion and pharmacological inhibition. Genetic ablation results in sustained loss of CD73 expression throughout tumor development. In contrast, antibody-based blockade primarily inhibits extracellular enzymatic activity and may be limited by factors such as tissue penetration, pharmacokinetics, and incomplete target engagement in vivo. Additionally, genetic deletion eliminates CD73 from tumor onset, whereas therapeutic blockade is initiated after tumor establishment, which may further contribute to the observed differences in efficacy.

Importantly, CD8^+^ T cell depletion completely abrogated the antitumor efficacy of the olaparib + α-CD73 combination (Figure 4K and Supplemental Figure 7J), whereas CD4^+^ T cell depletion had no effect (Supplemental Figure 7, K and L), indicating that CD8^+^ T cells, rather than CD4^+^ T cells, are indispensable for therapeutic benefit.

Despite the widespread baseline expression of CD73 in normal tissues, we observed no significant differences in plasma alanine aminotransferase and aspartate aminotransferase levels or body weight among the vehicle, olaparib, anti-CD73 single-agent, and combination treatment groups (Supplemental Figure 7, M–O), suggesting that the therapeutic regimen is well tolerated. Nevertheless, more comprehensive toxicity assessment — particularly for combination therapy — is required in future studies.

### Combination therapy enhances antitumor CD8^+^ T cell function and directs spatially immunocompetent interactions among orthotopic prostate tumors.

In accordance with the flank tumor studies, the superior antitumor effect of the olaparib + α-CD73 combination was confirmed in an orthotopic MycCaP prostate tumor model compared with control or monotherapy groups (Figure 5, A and B). Flow cytometry analysis revealed that olaparib treatment significantly increased surface CD73 expression on CD45^–^ tumor cells, whereas α-CD73 therapy reversed the olaparib-mediated adaptive upregulation of CD73 (Figure 5B). Olaparib did not significantly alter CD73 levels on major immune subsets (e.g., CD8^+^ T cells, Tregs, TAMs, and MDSCs) (Supplemental Figure 8A), supporting a tumor-intrinsic mechanism of CD73 induction. Further immunophenotyping showed increased CD4^+^ T cell infiltration with combination therapy, while the frequencies of PMN-MDSCs, monocytic-MDSCs (M-MDSCs), and TAMs remained unchanged (Supplemental Figure 8, B and C). Importantly, PD-L1 expression was reduced on both PMN-MDSCs and M-MDSCs following combination treatment, although not on TAMs (Supplemental Figure 8D). Notably, the combination also significantly increased CD8^+^ T cell infiltration and enhanced effector cytokine production (IFN-γ and TNF-α), while reducing the proportion of PD-1^+^ and CD39^+^ exhausted-like CD8^+^ T cells (Figure 5C).

Multiplex immunohistochemistry (mIHC) analyses corroborated these findings, demonstrating higher CD8^+^ T cell density and increased IFN-γ^+^ CD8^+^ T cells within tumor tissues following combination therapy (Figure 5, D–F). Spatial analysis further demonstrated that CD8^+^ T cells were positioned farther from CD73-expressing EpCAM^+^ tumor cells than from CD73-null tumor cells, indicating that CD73 expression may restrict effector T cell engagement. Importantly, combination therapy significantly reduced the distance between CD8^+^ T cells and CD73-expressing tumor cells (Figure 5G), suggesting improved immune accessibility. In addition, the number of proximal CD8^+^ T cells (within 20 μm) surrounding MHC-I–expressing EpCAM^+^ tumor cells was significantly increased in the combination group (Figure 5H), supporting enhanced tumor cell recognition by tumor-reactive CD8^+^ T cells. Consistent with flow cytometry findings, mIHC showed reduced CD73 expression but increased MHC-I expression on EpCAM^+^ tumor cells upon combination therapy (Supplemental Figure 8E), further corroborating improved antitumor T cell effector function and overall therapeutic efficacy.

To examine spatial gene expression within the heterogeneity of treated orthotopic MycCaP tumors, we performed spatial transcriptomic (ST) analysis using 10x Genomics Visium. Gene expression profiles of 13,193 barcoded ST spots across tumor sections were analyzed using unbiased Louvain clustering and t-SNE/UMAP algorithms (Figure 6, A–C). Six distinct clusters (C0–C5) were identified based on transcriptional patterns, reflecting intratumoral heterogeneity (Figure 6B). Combination-treated samples exhibited a modest increase in clusters C1 and C3 and a decrease in C4 compared with controls (Figure 6D). Differentially expressed genes within each cluster were subjected to Gene Ontology (GO) enrichment analysis. Upregulated genes were primarily detected in C1, C2, and C3, whereas downregulated genes were enriched in C0, C4, and C5 (Figure 6E and Supplemental Figure 9A). Notably, C3 was enriched for immune activation pathways, including APP, ER-phagosome, innate immune signaling, cytokine signaling, Toll-like receptor cascades, and IRF3/IRF7 activation. C1 also contained immune-related pathways such as IRAK2-mediated TAK1 activation upon TLR7/8/9 stimulation, ER-phagosome, and APP. In contrast, C4 exhibited marked downregulation of antigen presentation, late endosome microautophagy, and immune system pathways compared with other regions (Figure 6E). Immune-related pathways were largely absent in C0, C2, and C5. Signature gene sets for APP, IFN-γ signaling, and type I IFN response were preferentially enriched in C3 and C1 (Supplemental Figure 9B), indicating that C1 and C3 represent immunocompetent areas, whereas C4 corresponds to immunosuppressed regions. Consistent with this interpretation, combination-treated sections displayed abundant CD8^+^ T cell and APP^+^ spots within immunocompetent clusters (C1+C3), whereas immunosuppressive regions contained few CD8^+^ T cells (Figure 6, B and D). To validate these findings, we applied region segmentation analysis (100 μm radius) integrating gene expression, spatial coordinates, and histology. Spatial gene correlation identified 5 distinct gene modules (Figure 6F). Region gene module 5 (G5), enriched for APP, IFN-γ signaling, and type I IFN response pathways (Supplemental Figure 9C), aligned predominantly with C3 and, to a lesser extent, C1 (Figure 6G), confirming their immunocompetent identity. Notably, G5 also contained CD3^+^ T cell signatures and was preferentially distributed in combination-treated samples, supporting enhanced formation of immunocompetent regions across tumor tissues following olaparib plus CD73 blockade therapy.

To relate these patterns to tissue architecture, H&E-stained tumor sections were analyzed using the SpatialX imaging segmentation algorithm (Figure 6H). G5, and to a lesser extent G3, were notably enriched in tumor cores relative to reactive stroma and were preferentially detected in combination-treated tumors (Figure 6I). Collectively, these data support the emergence of immunocompetent niches within tumor cores in response to combined olaparib and CD73 blockade.

### CD73 upregulation correlates with reduced CD8^+^ T cell infiltration and protumorigenic signaling pathways in human prostate cancer.

To evaluate the clinical relevance of CD73 expression and its association with T cell infiltration and TGF-β/AKT signaling, we performed mIHC for CD8, CD73, pAKT, SMAD4, and PanCK on a prostate cancer tissue microarray (Figure 7A). CD73 intensity in PanCK^+^ epithelial cells was markedly higher in malignant specimens compared with normal, normal-adjacent, or hyperplasia tissues (Figure 7B), in line with the established role of CD73 in cancer progression (19, 20). In contrast, CD8^+^ T cell infiltration was increased in hyperplasia samples but substantially reduced in malignant tumors (Figure 7C).

As expected from oncogenic AKT activation in advanced disease, pAKT levels in PanCK^+^ tumor cells were significantly elevated in malignant specimens (Figure 7D). Notably, CD73 expression levels were higher in SMAD4^+^ and pAKT^+^ tumor cells compared with their respective counterparts (Figure 7E), supporting a link between CD73 upregulation and TGF-β/AKT pathway activity in human prostate tumors.

We next examined the spatial relationship between CD73^+^ tumor cells and CD8^+^ T cells. CD8^+^ T cells were located at significantly greater distances from CD73^+^ tumor cells than from CD73^–^ tumor cells, and the overall CD8^+^ T cell–tumor cell distance increased progressively from normal tissue to hyperplasia and ultimately to malignant specimens (Figure 7F). These findings indicate that CD73 expression may be associated with a CD8^+^ T cell–excluded tumor architecture.

Finally, survival analysis revealed that prostate cancer patients with a high combined gene signature — including *NT5E*, *PTEN*, *TGFB1*, *ATR*, *AKT*, and *CHEK1* — had significantly poorer overall survival (Figure 7G), underscoring the potential clinical impact of pathways identified in our mechanistic studies.

## Discussion

In this study, we identify CD73 upregulation as a previously unrecognized adaptive response to olaparib in prostate cancer and demonstrate that CD73 serves as a critical modulator of tumor–immune cell interactions and therapeutic resistance. Through integrated transcriptomic, mechanistic, functional, and spatial analyses coupled with efficacy evaluation, we show that olaparib not only enhances tumor cell–intrinsic immunogenicity but also induces compensatory increases in CD73 expression through 2 parallel and nonredundant pathways: ATR/IRF1 and TGF-β/AKT/SP1. These findings provide mechanistic insight into how prostate cancer cells respond to DNA damage stress and highlight CD73 as a therapeutically actionable target to improve the efficacy of olaparib-based therapies.

Consistent with prior reports that PARPi activates the STING/IFN signaling pathway and enhances antigen presentation (49–52), we observed robust induction of MHC-I and type I IFN–related pathways following olaparib treatment. Functionally, this translated into increased cytokine production, enhanced CD8^+^ T cell activation, and improved tumor cell killing. These results support the concept that olaparib converts prostate cancer cells — traditionally poorly immunogenic — into a more immunostimulatory state.

However, our data reveal a concurrent counterregulatory increase in CD73, the rate-limiting ectonucleotidase responsible for generating extracellular adenosine, a potent immunosuppressive metabolite that impairs T cell activation, cytokine production, and cytotoxicity (53, 54). Prior studies have shown that genomic instability, TGF-β signaling, hypoxia, and oncogenic stress can upregulate CD73 (55–57). Here, we demonstrate that olaparib consistently induces CD73 expression across genetically diverse prostate cancer models — including MycCaP (HRR-proficient), PTEN-deficient MycCaP-PTEN–KO (HRR-compromised), and Trp53/Rb-deficient DKO tumors — highlighting CD73 upregulation as a conserved response to olaparib-mediated genotoxic stress rather than a model-specific artifact. Genetic depletion of *Parp1* recapitulated this effect, confirming that CD73 upregulation is a direct consequence of PARP1 loss of function.

Mechanistically, olaparib induces DNA damage, activates ATR signaling, and promotes IRF1-driven transcription of NT5E (CD73), consistent with earlier work describing ATR–IRF1 interactions in DNA damage responses (12). ATR inhibition partially, but not completely, reversed CD73 induction, indicating additional regulatory mechanisms.

We identified a second, independent pathway in which PARPi activates TGF-β/AKT/SP1 signaling. Both TGF-β and AKT are well-established regulators of CD73 expression across tumor cells (41, 58), and we showed that TGF-β blockade prevented olaparib-induced AKT phosphorylation, placing AKT downstream of TGF-β. Our data demonstrate that olaparib engages this axis to further promote CD73 upregulation. The cooperative suppression of CD73 expression with dual ATR and TGF-β inhibition confirms 2 parallel, nonredundant pathways controlling CD73 downstream of olaparib-induced DNA damage. This complex regulation may underlie variability in CD73 expression across tumors and explains why single-pathway inhibition cannot fully suppress CD73 upregulation. It also highlights the plasticity of prostate tumor cells in engaging multiple stress response circuits to sustain immune evasion.

This induction of CD73 may explain why the immune-stimulatory signature triggered by olaparib is insufficient to drive durable tumor regression in vivo. Although olaparib increases antigen presentation and enhances STING-mediated inflammatory signaling, the resultant CD73-driven adenosine signaling establishes an immunosuppressive barrier that limits effector CD8^+^ T cell infiltration and function. This duality — enhanced immunogenicity paired with increased immunosuppression — likely constrains the effectiveness of olaparib monotherapy and may contribute to the weak responses seen in PARPi + ICI clinical trials (16, 17).

By targeting the adenosine axis, CD73 blockade revealed the more immunogenic potential of olaparib. Combination therapy was superior in inhibiting tumor growth and increasing survival across all syngeneic prostate cancer models we examined, regardless of HRR status. Of note, α-CD73 monotherapy showed activity in HRR-compromised PTEN-KO models, indicating increased dependence on CD73 in genomically unstable tumors. However, the strongest antitumor efficacy consistently occurred with olaparib + α-CD73 dual treatment.

Mechanistically, combination therapy increased CD3^+^, CD4^+^, and CD8^+^ T cell infiltration; expanded Tpex and effector CD8^+^ T cells; boosted IFN-γ and TNF-α production; and reduced exhaustion markers such as TOX and PD-1. These findings align with prior observations that adenosine blockade reinvigorates effector T cells and enhance the therapeutic activity of anti–PD-1 and anti–CTLA-4 mAbs on tumor growth (59, 60). Importantly, the combination also decreased PD-L1 expression on PMN-MDSCs and increased CD40 expression on DCs, consistent with a shift toward a less immunosuppressive and more immunostimulatory phenotype. While we did not directly assess myeloid cell polarization or perform in-depth functional analyses of all myeloid subsets, these observed changes suggest that CD73 inhibition may reprogram the tumor microenvironment to better support T cell priming and activation (61, 62). Although our in vivo depletion experiments demonstrated that CD8^+^ T cells are the primary required effectors for tumor control — as CD8^+^ T (but not CD4^+^ T) depletion completely abrogated therapeutic efficacy — we cannot exclude the functional roles played by other immune populations. CD73 expressed by immune cells is known to play a substantial role in adenosine-mediated immunosuppression within the tumor microenvironment. Moreover, currently available CD73-targeting agents predominantly inhibit its enzymatic activity rather than downregulating its expression. Therefore, it remains plausible that targeting CD73 on immune cells could further enhance the efficacy of PARPi, and this possibility warrants future investigation.

Spatial mIHC analysis in mouse orthotopic tumors indicated that CD8^+^ T cells localized farther from CD73-expressing tumors, consistent with the finding that adenosine prevents T cells from accessing tumor nests (22, 63). CD73 blockade reduced these exclusion distances and increased CD8^+^ T cell proximity to both CD73^+^ and MHC-I^+^ EpCAM^+^ tumor cells upon olaparib treatment, suggesting improved tumor cell recognition and engagement by tumor-reactive T cells. ST analysis using the standard Visium platform lacks single-cell resolution. Therefore, integrating deconvolution approaches with matched scRNA-seq data is required to enable spatial neighborhood analysis and validate the relationship between predicted cellular composition and histological features. Notably, immunocompetent regions — defined by spatial gene expression profiles — were primarily observed in tumor sections receiving combination therapy. These regions were enriched for pathways including APP, IFN-γ signaling, and type I IFN response, along with T cell signature genes, supporting the role of CD73 blockade in reinvigorating antitumor T cell activity upon olaparib treatment across the finer partitions of the tumor tissues. In human prostate tumors, CD73 was strongly upregulated in malignant epithelium but less so in normal or hyperplastic prostate tissue, and CD8^+^ T cells were spatially far from CD73-expressing tumor cells, indicating an immune-excluded and immunosuppressive tumor microenvironment, consistent with prior observations that CD73 expression in the prostate epithelium limits CD8^+^ T cell–mediated immunosurveillance (26). Furthermore, CD73 was preferentially expressed in SMAD4^+^ and pAKT^+^ tumor cells, consistent with the activation of the TGF-β/AKT pathway observed in vitro. Importantly, patients with high combined expression of *NT5E* (CD73), *PTEN*, *TGFB1*, *ATR*, *AKT*, and *CHEK1* had significantly poorer overall survival, underscoring the clinical importance of these pathways in aggressive prostate cancer. Our findings appear to conflict with a published report (26) that high CD73 expression in normal adjacent epithelium — not tumor epithelium — was associated with poorer patient survival, whereas elevated CD73 in the tumor stroma correlated with improved survival in univariate analysis. Notably, that study did not directly interrogate the contribution of stromal CD73 to immune evasion, a mechanism that has since been extensively explored (64, 65). In contrast, our study focuses on a distinct and treatment-specific context, namely, the adaptive response of prostate cancer cells to PARPi. Also, our study integrates CD73 within a broader regulatory network through a 6-gene signature (*NT5E*, *PTEN*, *TGFB1*, *ATR*, *AKT*, and *CHEK1*). This systems-level approach captures pathway activation rather than static expression alone and is associated with worse clinical outcomes, supporting a negative prognostic role for CD73 in this specific molecular context. Thus, the functional impact of CD73 is highly context dependent, and future studies are warranted to further dissect the roles of cell type–specific CD73 expression and adenosine signaling in prostate cancer progression and therapeutic response.

Notably, while PARPi robustly upregulates CD73, baseline constitutive expression of this ectonucleotidase is present across our prostate cancer models. Consequently, the combination therapy likely targets both preexisting CD73-mediated immunosuppression and the additional upregulation induced by PARPi. While baseline CD73 expression may contribute to therapeutic response, our data demonstrate that PARPi further increases CD73 levels, consistent with an adaptive response to treatment. In line with our ex vivo coculture experiments, both genetic and pharmacological inhibition of CD73 sensitized tumors to olaparib in vivo and enhanced antitumor immune responses. Together, these findings support the notion that CD73 upregulation functionally limits PARPi-induced antitumor immunity, rather than simply reflecting independent or additive effects of parallel pathways.

PARPi induces multiple immunomodulatory pathways, raising the possibility that CD73 upregulation could represent a correlated bystander effect rather than a causal mediator of immune resistance. However, our data support a functional, at least partial, causal role for CD73 in this process. Specifically, in our ex vivo coculture experiments, either olaparib treatment or tumor cell CD73 KD increased IFN-γ and TNF-α production, enhanced CD25 expression, and improved the cytotoxic activity of tumor-reactive CD8^+^ T cells. Notably, CD73 KD further amplified the immunostimulatory effects of olaparib, suggesting that olaparib-induced CD73 upregulation acts as an adaptive resistance mechanism that limits tumor cell recognition and killing by tumor-reactive T cells. Consistent with these findings, genetic and pharmacological blockade of CD73 was sufficient to sensitize tumors to olaparib treatment in vivo and significantly enhance antitumor immune responses. These results argue against CD73 being a passive bystander and instead support its functional contribution to PARPi-induced immune resistance. We acknowledge that additional immunosuppressive pathways likely contribute to the overall resistance landscape. However, our data indicate that CD73 represents a key and actionable node whose inhibition is sufficient to substantially restore antitumor immunity in the context of PARPi.

Our study provides compelling evidence that CD73 upregulation represents a universal adaptive response to PARPi in prostate cancer, occurring consistently in both HRR-compromised and -proficient tumors. This finding has important clinical implications, as fewer than 30% of mCRPC patients harbor HRR mutations and currently qualify for PARP inhibitor therapy. We demonstrate that combining PARP inhibitors with CD73 blockade enhances antitumor immunity in HRR-proficient tumors and may be particularly effective in cases with PTEN loss (present in 40%–60% of mCRPC), thereby expanding the therapeutic potential of this approach to the majority of mCRPC patients.

The absence of patient samples obtained following PARPi therapy represents a limitation of our study, largely due to the unavailability of such specimens. Nonetheless, our mechanistic investigations across in vitro and in vivo models consistently demonstrate that PARPi treatment induces CD73 upregulation, which in turn fosters an immunosuppressive tumor microenvironment and restricts CD8^+^ T cell–mediated antitumor immunity. These results provide a strong biological rationale to investigate whether a similar adaptive CD73-mediated resistance mechanism occurs in patients following PARPi therapy.

Future work should explore the upstream triggers of TGF-β activation following PARPi and evaluate the interplay with cGAS/STING signaling in modulating CD73. Given clinically available PARPi and anti-CD73 mAb/inhibitors, our research certainly opens the opportunity for the rapid translation of these preclinical findings into the clinic and shed new light on the development of more effective immune-based treatments for advanced prostate cancer. Additionally, developing biomarkers based on CD73 expression with spatial immune architecture may help stratify patients most likely to benefit. Collectively, this study uncovers CD73 as a DNA damage–inducible resistance mechanism to PARPi regulated by ATR/IRF1 and TGF-β/AKT/SP1 pathways. Therapeutic targeting of CD73 in combination with PARP inhibitors promotes CD8^+^ T cell–mediated antitumor immunity and suppresses tumor growth across HRR-proficient and -compromised prostate cancers, offering a promising strategy to broaden the clinical impact of PARP inhibitors in mCRPC. Although clinical responses to PD-1/PD-L1 inhibitors in prostate cancer have generally been limited, the combination of PARPi and CD73 blockade may help overcome the immunologically “cold” nature of this tumor. Therefore, investigating triple combination strategies that incorporate PD-1/PD-L1 blockade represents a rational and promising direction for future studies.

## Methods

### Sex as a biological variable.

The murine system used in our study was exclusively male because prostate cancer is only relevant in males.

### Mice and cell lines.

Eight-week-old male FVB mice or C57BL/6 mice were purchased from The Jackson Laboratory and housed at the Northwestern University Center for Comparative Medicine. All animal procedures were performed in accordance with protocols approved by the IACUC of Northwestern University.

The prostate cancer cell lines MycCaP, DU145, and PC3 were procured from ATCC. The MycCaP-PTEN–KO cells have been described previously (66). DKO (*Rb1*/*Trp53* KO) cells (37) were provided by David Goodrich (Roswell Park Comprehensive Cancer Center, Buffalo, New York, USA). All cell lines were maintained in high-glucose DMEM supplemented with 10% FBS and antibiotics (penicillin at 100 μg/mL and streptomycin at 100 μg/mL).

The CRISPR/Cas9 system was used to generate CD73-, Parp1-, and IRF-1–KO/KD cell lines in MycCaP cells. First, MycCaP cells were infected with Cas9-hygromycin lentiviral particles (Sigma-Aldrich) and selected using hygromycin B (400 μg/mL) for 2 weeks to establish a stable Cas9-expressing line. Following selection, 2 different sgRNAs per target gene (*Nt5e*, *Parp1*, or *Irf-1*), along with a nontarget sgRNA (Horizon Discovery), were introduced into the MycCaP-Cas9 cells via electroporation. The specific sgRNA sequences used were as follows: *Nt5e* target 1: TTACAATGCACATGGACACG; *Nt5e* target 2: ACCTCCGTTTACAATGCACA; *Parp1* target 1: CTGTGTCTTGACCATGCACT; *Parp1* target 2: AGGCCACCTCGATGTCCAGG; *Irf-1* target 1: GCTGGGCCATTCACACAGGT; and *Irf-1* target 2: GTGTAACTGCTGTGGTCATC.

### Bulk RNA-seq.

MycCaP cells were treated with 20 μM olaparib for 24 h prior to harvest. Total RNA was extracted using the Direct-zol RNA Miniprep Kit (Zymo Research) according to the manufacturer’s protocol, and RNA concentration was measured with the Qubit RNA High-Sensitivity Assay (Thermo Fisher Scientific). RNA integrity was verified using an Agilent 2100 Bioanalyzer. mRNA was enriched using the NEBNext Poly(A) mRNA Magnetic Isolation Module (New England Biolabs), and strand-specific cDNA libraries were generated using the NEBNext Ultra II RNA Library Prep Kit and NEBNext Multiplex Oligos for Illumina (New England Biolabs). AMPure XP beads (Beckman Coulter) were used for all RNA and DNA cleanup steps. Final library quantity and quality were assessed using the Qubit dsDNA High-Sensitivity Assay (Thermo Fisher Scientific). Sequencing was performed at the Northwestern University NUSeq Core on an Illumina HiSeq 4000 platform using single-end 50 bp reads, targeting approximately 20 million reads per sample.

Bulk RNA-seq data were processed for quality control using FastQC and Trimmomatic, followed by read mapping to the mm39 reference genome and quantification with STAR (https://github.com/alexdobin/STAR/). Differential expression analysis between the olaparib-treated and control groups was conducted using DESeq2 (https://bioconductor.org/packages/3.0/bioc/html/DESeq2.html). To interpret the biological significance of the results, functional enrichment and reactome pathway analyses were performed via DAVID (Database for Annotation, Visualization and Integrated Discovery) (https://davidbioinformatics.nih.gov), while broader transcriptional shifts were assessed through GSEA software (version 4.3.2) (https://www.gsea-msigdb.org/).

### Tumor cell–CD8^+^ T cell coculture assays.

MycCaP tumor cells were seeded in 6-well plates and treated with olaparib (20 μM) or vehicle control for 16 h. In parallel, CD8^+^ T cells were isolated and purified from the spleens of MycCaP tumor–bearing mice using magnetic bead–based separation. Following olaparib treatment, tumor cells were washed and cocultured with purified CD8^+^ T cells at a T cell/tumor cell ratio of 1:10. After 72 h of coculture, CD8^+^ T cells were collected and analyzed for activation markers and cytokine production by flow cytometry.

For cytotoxicity assays, MycCaP tumor-reactive effector CD8^+^ T cells were prepared as described previously (67, 68). These tumor-reactive CD8^+^ T cells were then cocultured with MycCaP tumor cells at a 1:1 effector/target ratio for 24 hours. Tumor cells were subsequently harvested and assessed for apoptosis by flow cytometry.

### Subcutaneous prostate tumor model establishment.

MycCaP or MycCaP-PTEN–KO cells (FVB background; 1 × 10^6^ cells) or DKO cells (C57BL/6 background; 1 × 10^6^ cells) were injected subcutaneously into the flank of mice on day 0. Tumor growth was monitored every 2–4 days by measuring 3 perpendicular diameters (a, b, and c), and tumor volume was calculated using the formula (a × b × c) / 2. Tumors exceeding 1,500 mm³ were considered to have reached endpoint criteria and were included in survival analyses.

### Orthotopic prostate cancer model.

Male FVB mice (6–8 weeks old) were anesthetized using isoflurane inhalation, and body temperature was maintained at 37°C throughout the procedure. Following disinfection of the lower abdomen, a small midline incision was made to expose the prostate gland. Using a 30-gauge insulin syringe, 10 μL of the MycCaP cell suspension (1 × 10^6^ cells mixed 1:1 with Matrigel in PBS) was carefully injected directly into the anterior prostate lobe. Care was taken to prevent cell leakage and ensure accurate placement. After the prostate was returned to the peritoneal cavity, the muscle layer and skin were closed with sutures and staples, respectively. Mice received postoperative analgesia and were monitored until full recovery, followed by regular observation and therapeutic treatments.

### Therapeutic treatments.

Treatment was initiated around day 7 postinoculation, when both subcutaneous tumors and, based on preliminary studies, the internal orthotopic tumors were estimated to have reached an approximate volume of 100 mm^3^. Mice were treated via i.p. injection in 4 distinct groups: olaparib at 10 mg/kg bodyweight daily, anti-CD73 monotherapy biweekly at 150 mg per mouse, combination therapy (olaparib + anti-CD73), or control (vehicle/isotype control IgG), as depicted in the experimental schematics. At study endpoint, mice were euthanized, and tumors were excised and weighed. For histological analysis, the central 3 mm thickness of each tumor was cut, embedded in OCT compound, and stored at –80°C. The remaining tumor tissue was immediately processed into single-cell suspensions by enzymatic digestion with collagenase/DNase followed by filtration through 70 μm strainers for subsequent flow cytometry analysis. For CD4/CD8 T cell depletion, mice received i.p. injections of α-CD8 (clone 2.43, Bio X Cell) or α-CD4 (clone GK1.5, Bio X Cell) at 150 μg/mouse. Injections started 3 days before tumor inoculation and continued every 3–4 days.

### ST analysis.

ST analysis was performed using the 10x Genomics Visium Spatial Gene Expression platform following the manufacturer’s instructions. Fresh-frozen MycCaP tumor tissues collected from mice treated with vehicle, olaparib, anti-CD73, or the combination therapy were embedded in OCT compound and cryosectioned at 10-μm thickness. Tissue sections were mounted onto Visium Spatial Gene Expression slides containing spatially barcoded capture areas and stored at −80°C until processing. Sections were fixed in cold methanol, stained with H&E, and imaged using a high-resolution bright-field microscope to enable spatial alignment and tissue annotation.

Following imaging, tissue sections were permeabilized for optimized durations to release mRNA, which was captured by spatially barcoded oligonucleotides on the slide surface. On-slide reverse transcription was performed to generate spatially indexed cDNA, followed by second-strand synthesis, cDNA amplification, and library construction according to the Visium protocol. Sequencing libraries were quality controlled and sequenced on an Illumina (NovaSeq X Plus) platform to generate paired-end reads.

Raw sequencing data were processed using the 10x Genomics Space Ranger pipeline for read alignment to the mouse reference genome, spatial barcode assignment, and gene expression quantification. The resulting spatial gene expression matrices were integrated with corresponding histological images. Downstream analyses, including normalization, clustering, differential gene expression, and spatial visualization, were conducted using the SpatialX platform from BioTuring. Spatial feature mapping and pathway-level analyses were performed to assess treatment-associated transcriptional changes within defined tumor regions. To address the relationship between ST clusters and tissue architecture, H&E-stained sections of treated tumors were analyzed using the SpatialX imaging segmentation active learning algorithm. This approach enabled segmentation of tissue into tumor core/nest (*Psca*, *Ar*, *Myc*) and reactive stroma (*Fgf1*, *Col1a1*, *Tnc*) based on cellular compartment–specific hallmark features, with validation by a pathologist.

### Disease-free survival analysis.

Disease-free survival analysis was performed using the GEPIA2 (Gene Expression Profiling Interactive Analysis 2) online tool (http://gepia2.cancer-pku.cn/#index). The analysis utilized a multigene signature comprising *NT5E*, *PTEN*, *TGFB1*, *ATR*, *AKT*, and *CHEK1*. Within the prostate cancer dataset (TCGA/GTEx data available within GEPIA2), the combined expression profile of these genes was used for survival stratification. Patients were dichotomized into high- and low-expression groups using the median expression value of the signature as the cutoff threshold. A Kaplan-Meier survival plot was generated, and the HR, along with the 95% CI and *P* value, were calculated using the log-rank test to assess the difference in disease-free survival between the high- and low-expression groups.

Additional details on the methods used in this study can be found in Supplemental Methods.

### Statistics.

Data are presented as mean ± SEM unless otherwise noted. Statistical analyses were conducted using GraphPad Prism (v10). Significance was assessed via unpaired 2-tailed Student’s *t* test or 1- or 2-way ANOVA, followed by Tukey’s post hoc test for multiple comparisons. Survival and tumor-free intervals were compared using the log-rank (Mantel-Cox) test. Correlations between variables were examined using either Pearson’s or Spearman’s methods, with *P* values < 0.05 considered statistically significant.

### Study approval.

All animal experiments were approved by the IACUC of Northwestern University (IS00007168).

### Data availability.

The bulk RNA-seq data (GSE312794), and Visium data (GSE315487) generated in this study have been uploaded to the Gene Expression Omnibus and will be publicly available as of the date of publication. This article does not report any original code. All functionality used to analyze the sequencing data is publicly available via the software packages described in Methods. Values for all data points in graphs are reported in the Supporting Data Values file.

## Author contributions

Conception and design: PX, RM, MZ, and BZ. Development of methodology: PX, RM, MZ, JF, LS, and BZ. Acquisition of data (provided animals, acquired and managed patients, provided facilities, etc.): PX, RM, MZ, JF, HT, and LS. Analysis and interpretation of data (e.g., statistical analysis, biostatistics, computational analysis): PX, RM, MZ, and BZ. Writing, review, and/or revision of the manuscript: PX, RM, MZ, JF, HT, LS, YW, TMK, DF, WC, JDW, SAA, YZ, AP, and BZ. Administrative, technical, or material support (i.e., reporting or organizing data, constructing databases): PX, JF, and BZ. Study supervision: BZ.

## Conflict of interest

The authors have declared that no conflict of interest exists.

## Funding support

This work is the result of NIH funding, in whole or in part, and is subject to the NIH Public Access Policy. Through acceptance of this federal funding, the NIH has been given a right to make the work publicly available in PubMed Central.

- NIH CA290743 (to BZ).
- NIH CA258857 (to YW and BZ).
- SPORE funding, P50CA180995 (to BZ, AP, and SAA).
- Cancer Center Support Grant (P30CA060553) to the Immunotherapy Assessment Core Facility at Robert H. Lurie Comprehensive Cancer Center of Northwestern University.

## Supplementary Material

Supplemental data

Unedited blot and gel images

Supporting data values

## Figures and Tables

**Figure 1:**
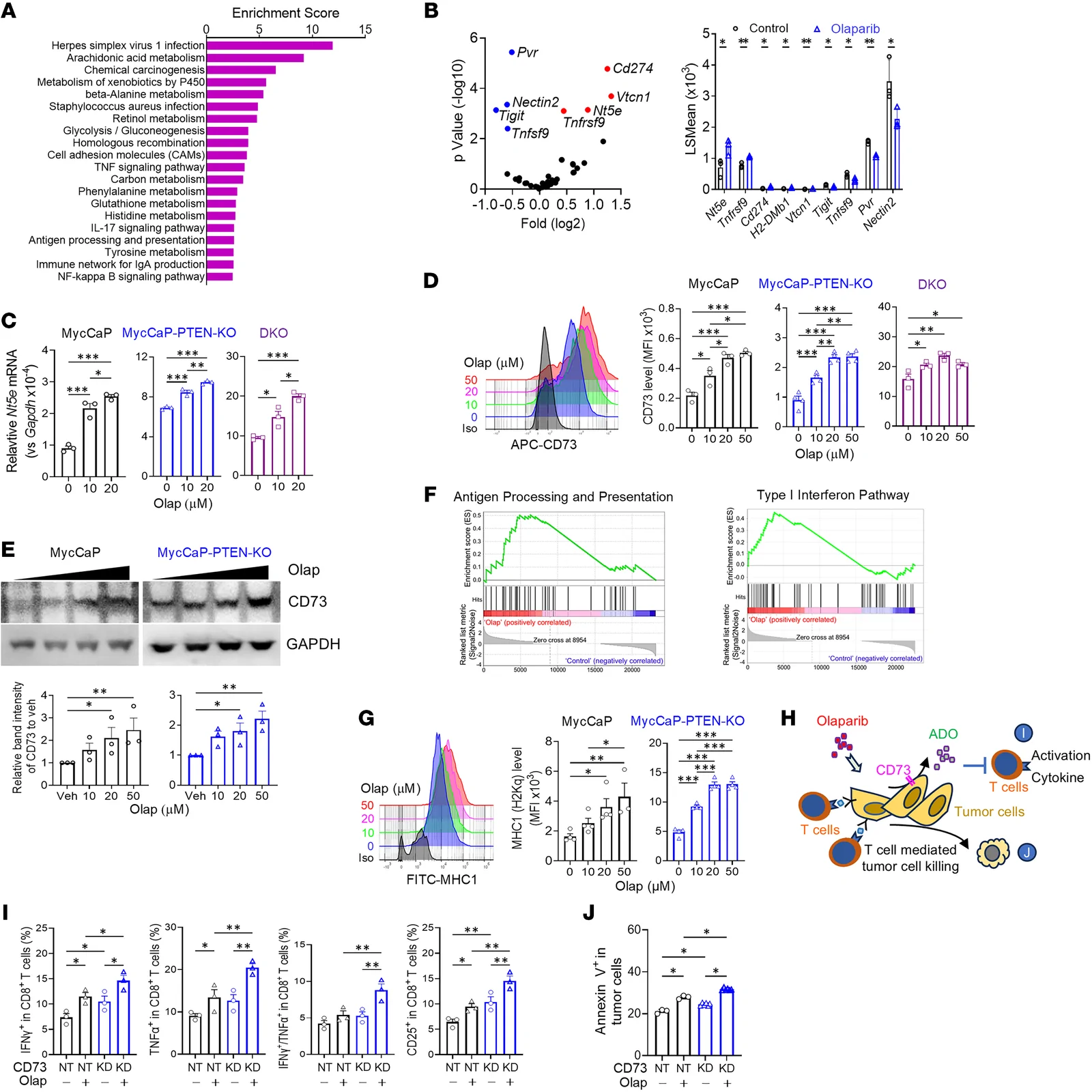
PARPi induces CD73 upregulation in prostate cancer cells, conferring adaptive immune resistance. (**A**) Reactome pathway analysis of differentially expressed genes (DEGs) from bulk RNA-seq in MycCaP cells treated with olaparib compared with vehicle control, using the DAVID. (**B**) Olaparib treatment induced DEGs among 45 detected immune checkpoint–related molecules in bulk RNA-seq. (**C**) RT-qPCR analysis of induced Nt5e expression in MycCaP, MycCaP-PTEN–KO, and DKO cells following olaparib treatment. (**D**) Flow cytometry analysis of dose-dependent increases in surface CD73 levels following 24 h olaparib treatment in MycCaP, MycCaP-PTEN–KO, and DKO cells. (**E**) Western blot analysis of CD73 protein expression and quantification in MycCaP and MycCaP-PTEN–KO cells treated with olaparib (0, 10, 20, and 50 μM) for 24 h. (**F**) GSEA revealing enrichment of antigen-processing/ presentation and type I IFN–related pathways following olaparib treatment. (**G**) Flow cytometry analysis of MHC-I (H-2Kq) expression in MycCaP and MycCaP-PTEN–KO cells following olaparib treatment. (**H**) Schematic overview of coculturing pretreated nontargeting control or CD73-KD MycCaP with tumor-reactive CD8^+^ T cells. (**I**) Flow cytometry analysis of IFN-γ and TNF-α production, and CD25 expression in cocultured CD8^+^ T cells. (**J**) Measurement of tumor cell killing by cocultured CD8^+^ T cells. NT, nontargeting control. Results are representative of 2 (**A**–**D** and **J**) or 3 (**E**, **G**, and **I**) independent experiments. Data were analyzed by unpaired 2-tailed Student’s *t* test (**B**), 1-way ANOVA with Tukey’s post hoc correction (**C**–**E** and **G**), or 2-way ANOVA with Tukey’s post hoc correction (**I** and **J**). Data plotted are mean ± SEM from biological replicates. *P < 0.05, **P < 0.01, ***P < 0.001.

**Figure 2:**
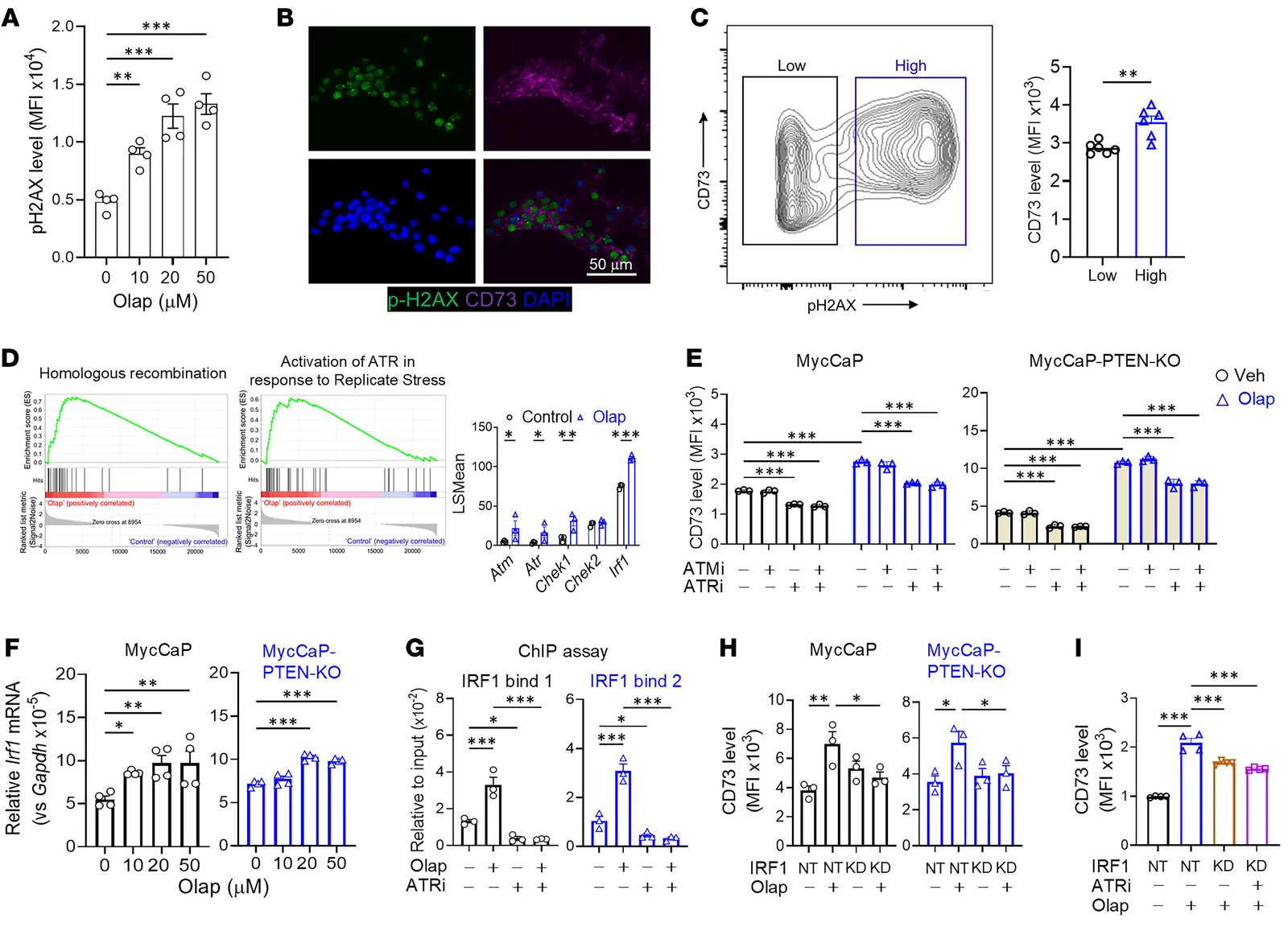
PARPi induces tumor CD73 expression through ATR–IRF1 signaling. (**A**) Flow cytometry analysis of p-H2AX levels in MycCaP cells following olaparib treatment. (**B**) Immunofluorescence staining showing spatial localization patterns of p-H2AX (green) and CD73 (purple) in MycCaP cells. Scale bar: 50 μm. (**C**) Flow cytometry assessment of the relationship between CD73 and p-H2AX signal intensity in MycCaP cells. (**D**) GSEA revealing enrichment of homologous recombination and ATR-associated pathways in MycCaP cells following olaparib treatment. (**E**) Flow cytometry analysis of CD73 expression in MycCaP and MycCaP-PTEN–KO cells pretreated with ATR inhibitor, ATM inhibitor, or the combination for 30 minutes before olaparib exposure. (**F**) RT-qPCR measurement of *Irf1* mRNA levels in MycCaP and MycCaP-PTEN–KO cells after olaparib treatment at 10, 20, or 50 μM. (**G**) ChIP analysis of IRF1 binding to 2 regions of the *Nt5e* promoter in MycCaP cells treated with olaparib, an ATR inhibitor, or the combination. (**H**) Flow cytometry assessment of CD73 levels in IRF1-KD versus nontargeting control (NT) MycCaP and MycCaP-PTEN–KO cells following olaparib treatment. (**I**) Flow cytometry analysis of CD73 expression in IRF1-KO MycCaP cells pretreated with an ATR inhibitor, followed by 20 μM olaparib treatment for 24 hours, compared with NT control MycCaP cells. Results are representative of 2 (**A**, **F**, and **G**) or 3 (**C**, **E**, **H**, and **I**) independent experiments. Data were analyzed by unpaired 2-tailed Student’s *t* test (**C** and **D**), 1-way ANOVA with Tukey’s post hoc correction (**A** and **F**), or 2-way ANOVA with Tukey’s post hoc correction (**E** and **G**–**I**). Data plotted are mean ± SEM from biological replicates. *P < 0.05, **P < 0.01, ***P < 0.001.

**Figure 3:**
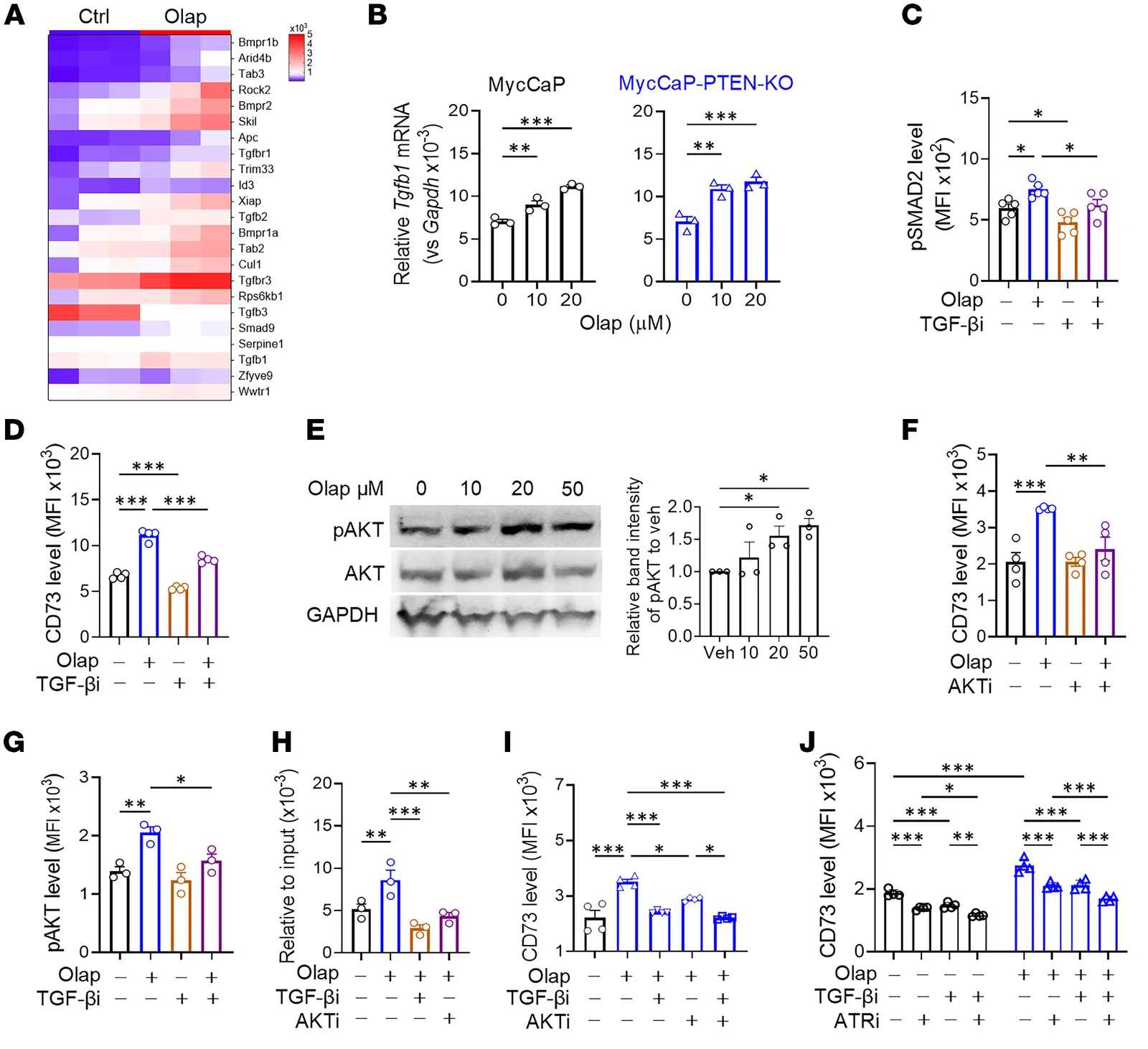
PARPi upregulates CD73 expression through the TGF-β/AKT signaling axis. (**A**) Heatmap presenting DEGs related to the TGF-β pathway in olaparib-treated MycCaP cells relative to control. (**B**) RT-qPCR measurement of *Tgf-b1* mRNA levels in MycCaP and MycCaP-PTEN–KO cells treated with 10 or 20 μM olaparib. (**C**) Flow cytometry analysis of pSMAD2 levels in MycCaP cells following olaparib treatment with or without a TGF-β inhibitor. (**D**) Flow cytometry assessment of CD73 expression in MycCaP cells treated with olaparib in the presence or absence of a TGF-β inhibitor. (**E**) Western blot analysis of pAKT levels in MycCaP cells treated with olaparib at indicated concentrations. (**F**) Flow cytometry analysis of CD73 expression in cells pretreated with an AKT inhibitor for 30 minutes before olaparib exposure. (**G**) Flow cytometry measurement of pAKT levels in MycCaP cells treated with olaparib with or without a TGF-β inhibitor. (**H**) Flow cytometry analysis of CD73 expression in MycCaP cells treated with olaparib in combination with a TGF-β inhibitor, an AKT inhibitor, or both. (**I**) ChIP assay showing SP1 binding to the Nt5e promoter in treated MycCaP cells. (**J**) Flow cytometry assessment of CD73 expression in MycCaP cells treated with or without olaparib in the absence or presence of a TGF-β inhibitor, an ATR inhibitor, or both. Results are representative of 2 (**B** and **G**) or 3 (**C**–**F** and **H**–**J**) independent experiments. Data were analyzed by 1-way ANOVA (**B** and **E**) or 2-way ANOVA (**C**, **D**, and **F**–**J**). Data plotted are mean ± SEM from biological replicates. *P < 0.05, **P < 0.01, ***P < 0.001.

**Figure 4:**
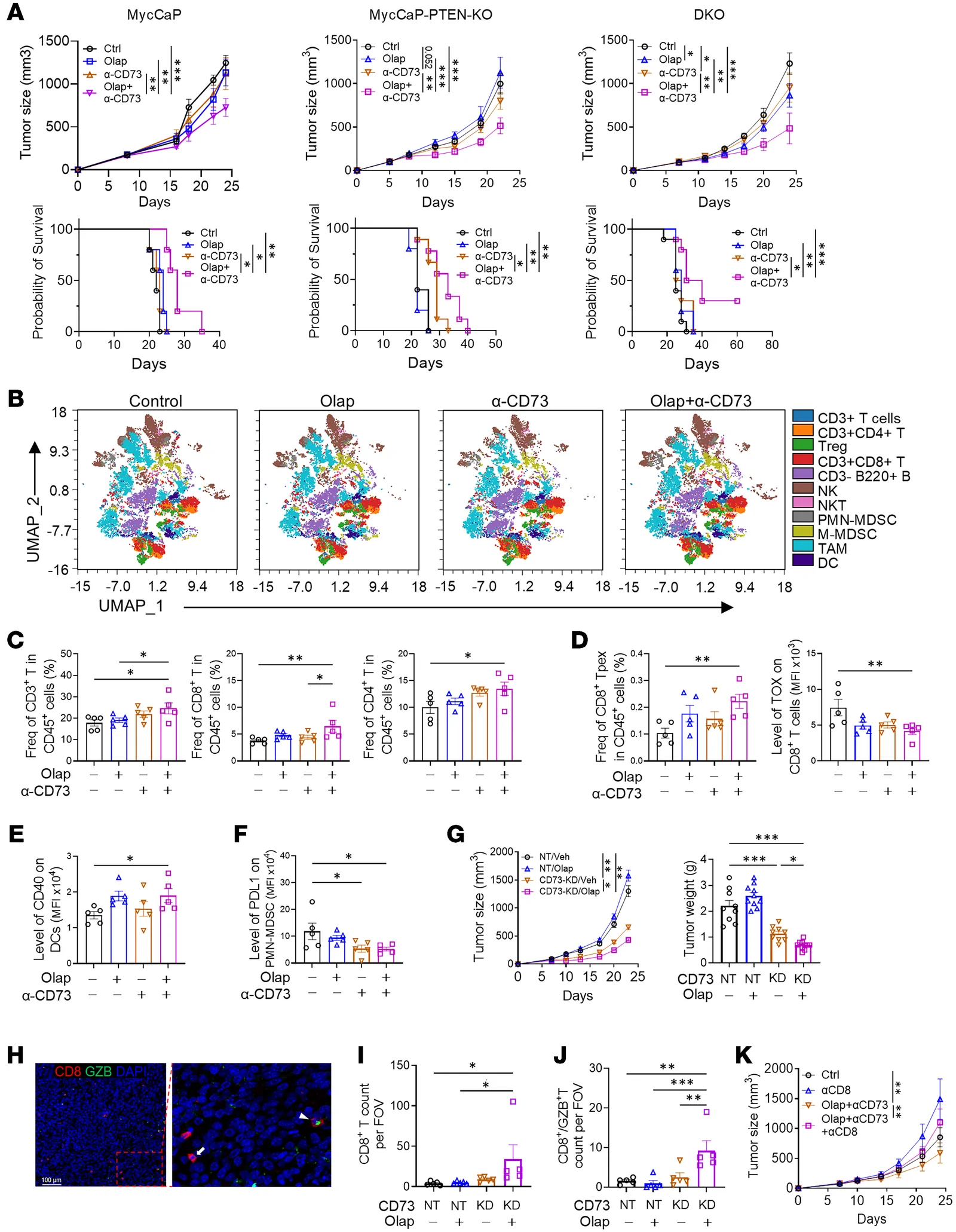
PARPi plus CD73 blockade substantially reduces tumor growth via CD8^+^ T cell–dependent mechanisms. (**A**) Syngeneic prostate cancer models (MycCaP, MycCaP-PTEN–KO, and DKO) were treated with vehicle, olaparib, α-CD73, or the combination (*n* = 5–10). Tumor growth kinetics (top) and survival curves (bottom) were recorded across treatment groups. (**B**) Tumor-infiltrating immune cells from each treatment group were analyzed by CyTEK and visualized using an unbiased UMAP analysis. (**C**) CyTEK analysis of the frequencies of CD3^+^, CD4^+^, and CD8^+^ T lymphocytes across treatment groups (*n* = 5). (**D**) Characterization of the Tpex subset and exhausted-like hallmark TOX expression in intratumoral CD8^+^ T cells (*n* = 5). (**E**) Expression of CD40 on tumor-infiltrating DCs across treatment groups (*n* = 5). (**F**) PD-L1 expression on PMN-MDSCs across treatment groups (*n* = 5). (**G**) Growth curves and tumor weights of MycCaP-NT and MycCaP-CD73–KD tumors treated with vehicle or olaparib (*n* = 9–10). (**H**–**J**) Representative immunofluorescence staining of CD8 and granzyme B (GZB) in MycCaP tumor sections (**H**), with quantification of CD8^+^ T cell density (**I**) and GZB^+^ CD8^+^ T cell density (**J**) across NT, olaparib, CD73-KD, and olaparib-treated control versus CD73-KD tumors (*n* = 5). White arrow, CD8^+^ GZB^–^ cells; white arrowhead, CD8^+^ GZB^+^ cells. Scale bar: 100 μm. (**K**) Tumor growth in mice treated with combined olaparib and α-CD73 following CD8^+^ cell depletion (*n* = 7–8). Results in **A** are representative of 3 independent experiments. Data were analyzed by 2-way ANOVA (**A**, **C**–**G**, and **I**–**K**) or log-rank test for survival curves (**A**). Data plotted are mean ± SEM from biological replicates. *P < 0.05, **P < 0.01, ***P < 0.001.

**Figure 5:**
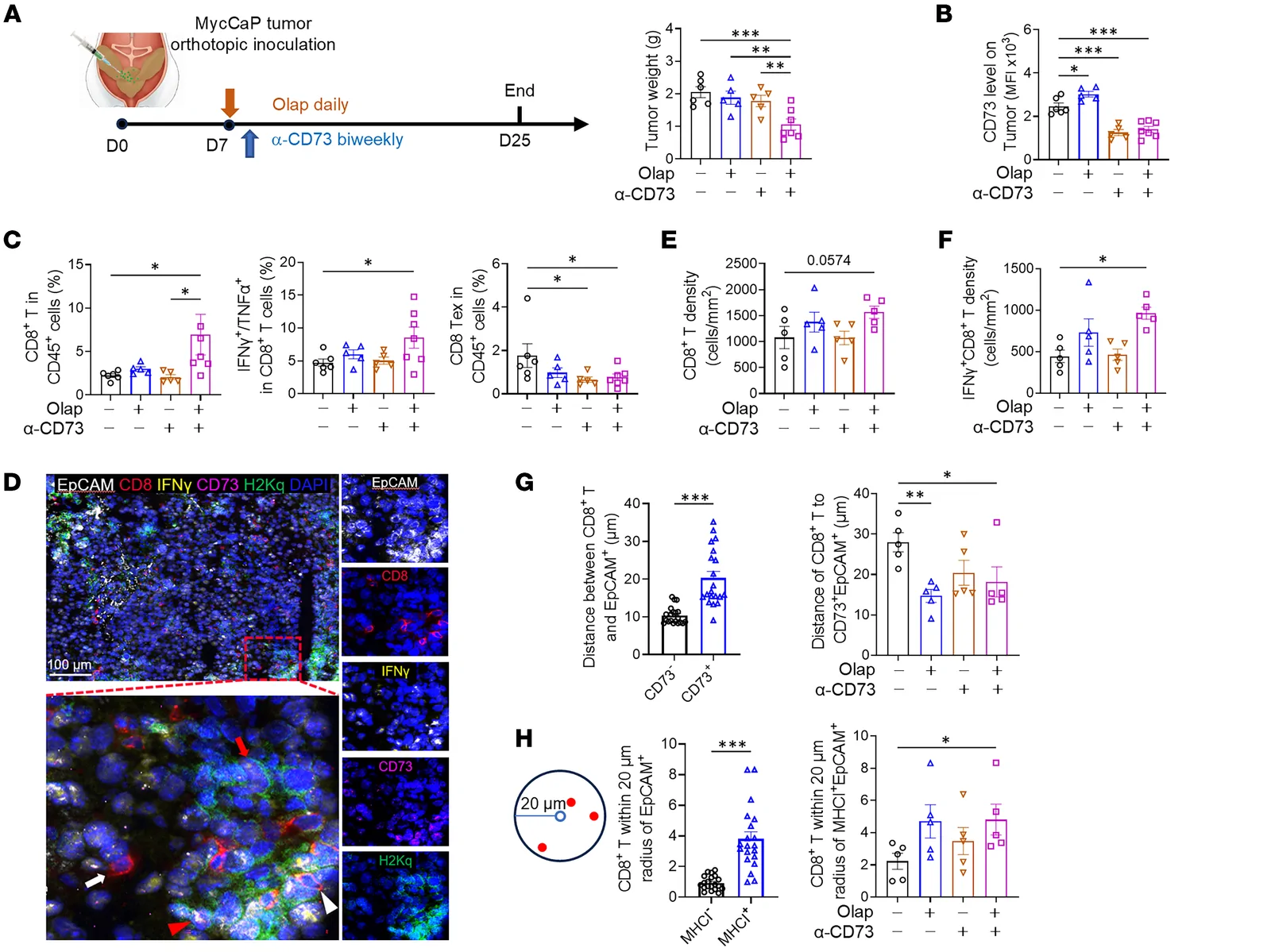
Combination therapy enhances antitumor CD8^+^ T cell function and directs spatially immunocompetent interactions among orthotopic prostate tumors. (**A**) Schematic of the orthotopic MycCaP prostate tumor model and treatment groups, including vehicle control, olaparib monotherapy, α-CD73 monotherapy, and combined olaparib plus α-CD73 (*n* = 5–7). The tumor weight analyzed at the end of the experiment. (**B**) Flow cytometry analysis of surface CD73 expression on EpCAM^+^ tumor cells from treated mice (*n* = 5–7). (**C**) Flow cytometry profiling of tumor-infiltrating CD8^+^ T cells, including total CD8^+^ T cell infiltration, IFN-γ and TNF-α expression, and proportions of exhausted-like CD8^+^ T cells (Tex; PD1^+^ and CD39^+^) (*n* = 5–7). (**D**) Representative mIHC analysis of EpCAM, CD8, IFN-γ, MHC-I (H2Kq), and CD73 staining in orthotopic MycCaP tumors. White arrow, CD8^+^ IFN-γ^–^ cells; white arrowhead, CD8^+^ IFN-γ^+^ cells; red arrow, EpCAM^+^ CD73^–/lo^ cells; red arrowhead, EpCAM^+^ CD73^+^ cells. Scale bar: 100 μm. (**E**) Quantification of CD8^+^ T cell density in tumor tissue by mIHC (*n* = 5). (**F**) Quantification of intratumoral IFN-γ^+^ CD8^+^ T cells density (*n* = 5). (**G**) Spatial analysis of the distance between intratumoral CD8^+^ T cells and EpCAM^+^ tumor cells stratified by positive or low/negative CD73 expression (*n* = 5). (**H**) Quantification of CD8^+^ T cells located within 20 μm of EpCAM^+^ tumor cells categorized by positive or low/negative MHC-I (H2Kq) expression (*n* = 5). Data were analyzed by 2-way ANOVA (**A**–**H**) or unpaired 2-tailed Student’s *t* test (**G** and **H**). Data plotted are mean ± SEM from biological replicates. *P < 0.05, **P < 0.01, ***P < 0.001.

**Figure 6:**
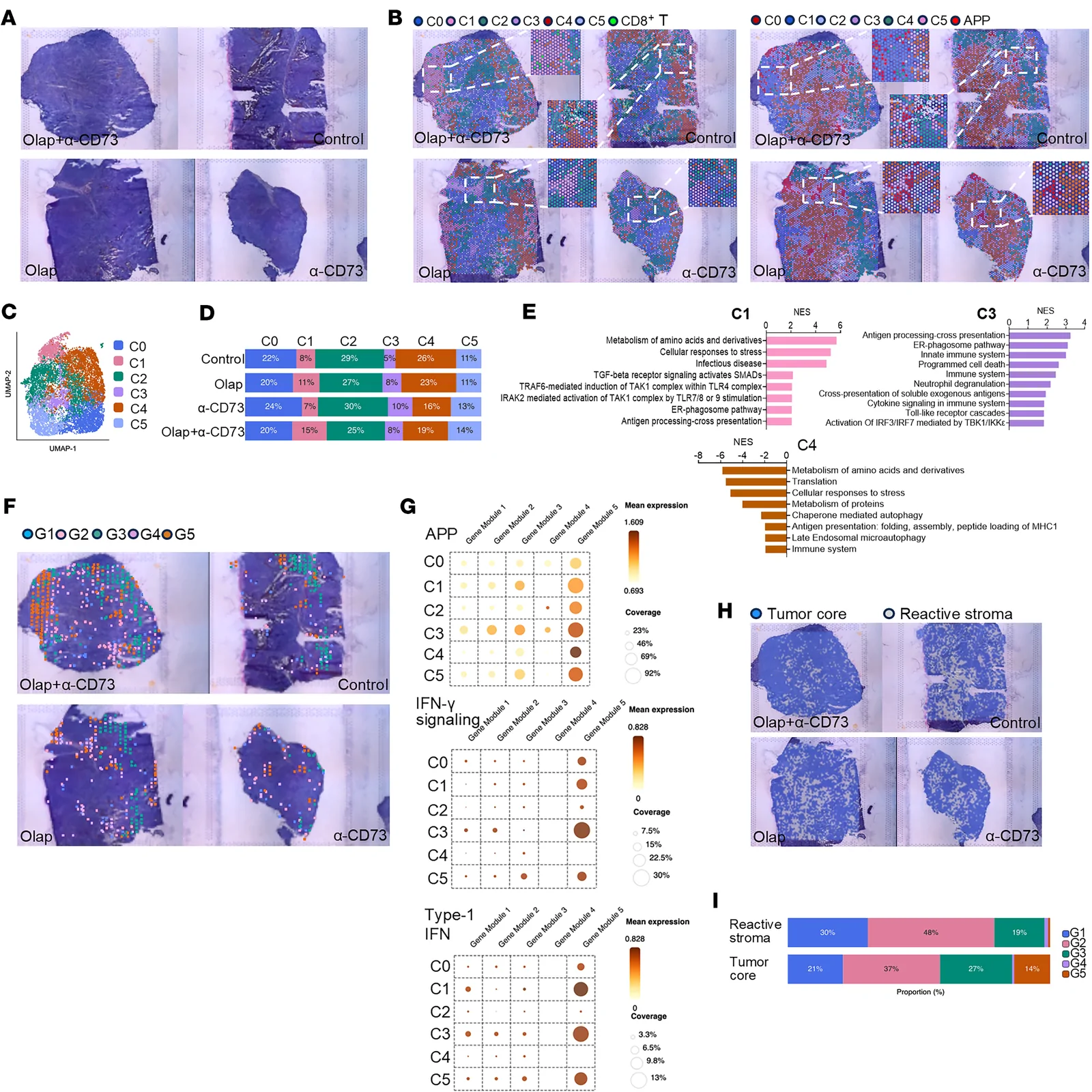
ST analysis reveals distinct immunocompetent regions associated with combination treatment. (**A** and **B**) Visualization of the 10x Genomics Visium results for treated orthotopic MycCaP tumor tissue sections. H&E staining (**A**) and 13,193 ST spots are presented (**B**). Treated tissues were clustered via unbiased Louvain clustering (resolution = 0.5), with different colors indicating manually defined CD8^+^ T cell regions (**B**, left). The areas with enriched APP signatures are visualized among each sample across clusters (**B**, right). (**C**) All 6 clusters were plotted according to UMAP algorithms. (**D**) The proportion of each cluster across samples. (**E**) Using SpatialX Software, the significantly upregulated and downregulated genes in each cluster were determined and subjected to GO enrichment analysis (Rectome). The top GO terms of each cluster are presented as bar graphs with normalized enrichment score (NES). (**F**) Region segmentation analysis (with a 100 μm radius) based on spatial gene correlation showing 5 distinct gene modules across samples. (**G**) Correlation of signature gene expression of APP, IFN-γ signaling, and type I IFN response between Louvain clusters and segmented gene modules. (**H**) Imaging segmentation analysis based on cellular compartment–specific hallmark features showing distinct tumor cores and reactive stroma compartments across H&E-stained samples. (**I**) The proportion of each spatial region identified in **F** across tumor cores and reactive stroma compartments.

**Figure 7:**
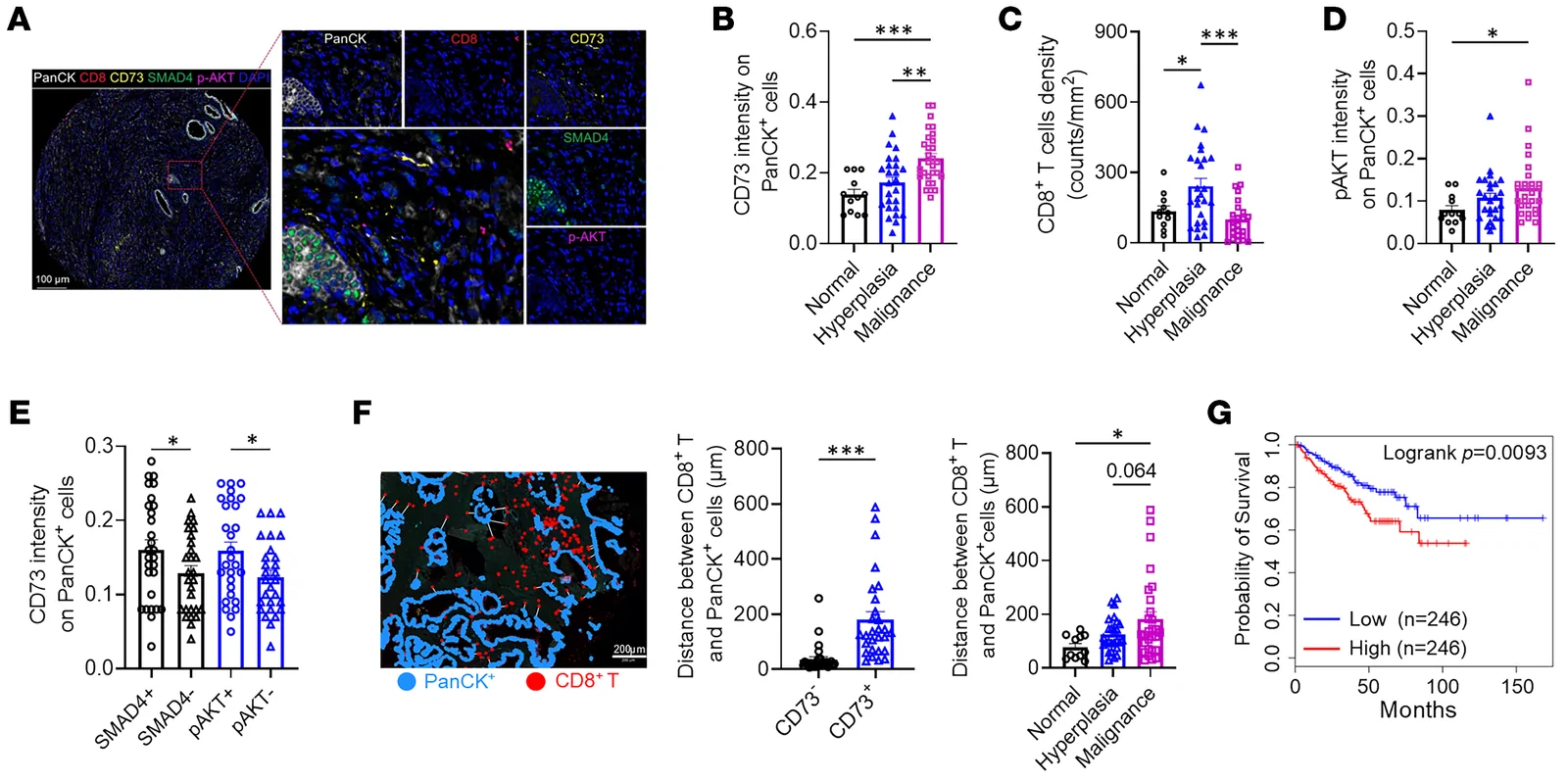
CD73 expression is associated with reduced CD8^+^ T cell infiltration and activation of TGF-β/AKT signaling in human prostate cancer. (**A**) Representative mIHC images of CD8, CD73, pAKT, SMAD4, and PanCK staining in human prostate cancer tissue microarray samples. Scale bar: 100 μm. (**B**–**D**) Quantification of CD73 intensity on tumor cells (**B**), CD8^+^ T cell infiltration (**C**), and tumor-associated pAKT levels (**D**) across normal/adjacent normal tissues (*n* = 12), hyperplasia lesions (*n* = 26), and malignant specimens (*n* = 28). (**E**) Comparison of CD73 expression on tumor cells stratified by SMAD4 status (SMAD4^+^ vs. SMAD4^–^) or pAKT status (pAKT^+^ vs. pAKT^–^) in malignant tissues. (**F**) Analysis of the distance between CD8^+^ T cells and PanCK^+^ tumor cells with CD73^+^ versus CD73^–^ staining and comparison of CD8^+^–tumor cell distances among normal, hyperplasia, and malignant tissues. Scale bar: 200 μm. (**G**) Survival curve analysis using a putatively defined gene signature comprising *NT5E*, *PTEN*, *TGFB1*, *ATR*, *AKT*, and *CHEK1* in prostate cancer patients. Data were analyzed by 1-way ANOVA (**B**–**D** and **F**) or unpaired 2-tailed Student’s *t* test (**E** and **F**). Data plotted are mean ± SEM from biological replicates. *P < 0.05, **P < 0.01, ***P < 0.001.

## Version 1 08/18/2026

In-Press Preview

## Version 2 10/01/2026

Electronic publication
