# Supplementary material for: CD73 inhibition overcomes adaptive immune resistance to PARP inhibition in models of prostate cancer
Source: J Clin Invest. 2026 Aug 18;136(19):e203785. doi: 10.1172/JCI203785 (PMC13626842; doi:10.1172/JCI203785)
Supplement: Supplementary file 1 [file jci-136-203785-s256.pdf]

Supplemental Methods

RNA extraction and quantitative Real-Time PCR. Total RNA was isolated using TRIzol reagent (Invitrogen) following the manufacturer's instructions. Complementary DNA (cDNA) was synthesized using the SuperScript One-Step RT-PCR Kit (Invitrogen). Quantitative real-time PCR (qRT-PCR) was performed using SYBR Green Master Mix (Bio-Rad) to quantify gene expression. Relative mRNA levels were calculated using the ΔΔCt method and normalized to GAPDH. The primers used for qRT-PCR were as follows: The primers used for q-RT-PCR are: GAPDH F: 5'- ACCACAGTCCATGCCATCAC-3', R: 5'-TCCACCACCCTGTTGCTGTA-3'; Nt5e F: 5′- CGCTCAGAAAGTTCGAGGTGTG -3′, R: 5′-CGCAGGCACTTCTTTGGAAGGT-3′; PARP1: F: 5'- AAGGCGGAGAAGACATTGGG -3', R: 5'- ACCATCTTCTTGGACAGGCG-3'; IRF1 F: 5'-TCCAAGTCCAGCCGAGACACTA-3', R: 5'- ACTGCTGTGGTCATCAGGTAGG-3'

Chromatin Immunoprecipitation (ChIP) assay. Chromatin immunoprecipitation was performed using the ChIPAb+ E2F-1 kit (Millipore) according to the manufacturer's instructions. Treated cells were first cross-linked with 1% paraformaldehyde for 10 minutes at room temperature and quenched with glycine. Cell pellets were lysed in SDS lysis buffer supplemented with protease inhibitors, and chromatin was fragmented by sonication. Sheared chromatin was incubated overnight with antibodies specific for IRF-1 (Millipore), SP1, or a normal IgG control. Immune complexes were captured using protein A/G beads, washed extensively, and eluted. Cross-links were reversed, and DNA was purified using the spin-column purification system included in the kit. ChIP-enriched DNA was analyzed by quantitative real-time PCR, and enrichment was calculated relative to input DNA.The primers used for ChIP assay were: IRF-1: forward 5′-GCCCTTCTACAATGCCAAAG-3′, reverse 5′-TTGTGATGGGTGTGAACCAC-3′; SP1: forward 5′- GAGAAATCGGTGTAATAGAGAGT -3′, reverse 5′- ACAGAGAGGAGGTTGTTCGC-3′; IRF-1 site1: forward 5′- GCAATGTTGACTGCCAGAGG -3′, reverse 5′- CCCGGCAGGAAAACAGTCT-3′; IRF-1 site2: forward 5′- CAGAACACACGGGAACCTCA -3′, reverse 5′-GCCACAGCACAAATTCCCTG -3′.

Western blotting. Cells were lysed in RIPA buffer (Sigma-Aldrich, R0278) containing protease and phosphatase inhibitors and incubated on ice for 30 minutes. After centrifugation (12,000 × g, 15 min, 4°C), protein concentrations were measured using the BCA assay (Pierce). Equal protein amounts were separated by SDS–PAGE and transferred to PVDF membranes using the Trans-Blot Turbo system (Bio-Rad). Membranes were blocked in 5% nonfat milk in TBS-T for 1 hour, incubated with primary antibodies overnight at 4°C, washed, and then incubated with HRP-conjugated secondary antibodies for 1 hour. Signals were developed using SuperSignal™ West Femto substrate and imaged on a Bio-Rad ChemiDoc™ MP system. GAPDH was used as the loading control. Western blot bands from independent experiments were quantified using ImageJ and normalized to the internal reference (GAPDH). The primary antibodies-anti-CD73 (Clone. D7F9A), anti-GAPDH (Clone. 14C10), anti-AKT (Clone. 11E7), anti-pAKT (Ser473) (Clone. D9E) were purchased from Cell Signaling Technology Inc. Anti-SP1 (PA5-29165) was purchased from ThermoFisher Scientific.

Flow cytometry analysis. To prevent non-specific antibody binding to Fc receptors, all single-cell suspensions were initially incubated with 2.4G2. Olaparib, talazoparib, VE-821 (ATR inhibitor), KU55933 (ATM inhibitor), SB525334 (TGFβ1 receptor inhibitor), M-2206 (AKT inhibitor), STING-IN-2 (STING inhibitor) were purchased from MedChemExpress Inc. Cells were treated with these inhibitors, then stained with 1 μg of the relevant monoclonal antibodies (mAbs) (Table 1), followed by two washes with cold PBS. For cell apoptosis analysis, cells were rinsed with 1X binding buffer and then stained simultaneously with Annexin V and 7AAD. Staining was performed in the binding buffer at room temperature (RT) in the dark for 10 minutes, and

samples were immediately acquired on the flow cytometer. Intracellular staining for IFN-γ, TNF-α in T cells was performed following manufacturer's instructions (BD Bioscience). Cells were stimulated with 50 ng/ml PMA, 5 μg/ml ionomycin, and 10 μg/ml BFA in the presence of the relevant tumor antigen peptides. Samples were acquired using the LSRII and FACSCanto flow cytometers. Data analysis was subsequently performed using FlowJo software or OMIQ.

Multiplex immunohistochemistry (mIHC). mIHC was performed on a prostate tissue microarray containing 80 malignant, hyperplastic, normal, and adjacent normal samples (US Biomax). Staining followed the Opal multiplex protocol (AKOYA Biosciences) according to previously published protocols. After deparaffinization and AR9 antigen retrieval, five iterative staining cycles were performed using primary antibodies against CD8, CD73, pAKT, SMAD4, and PanCK, with HRP-conjugated secondaries and distinct Opal fluorophores (Table 2). AR6 buffer was used between cycles to strip antibodies without removing fluorophores. Slides were counterstained with DAPI, and spectral unmixing was performed using single-marker controls. Additionally, paraffin sections of CD73-KO MycCaP tumor tissues treated with Olaparib were subjected to CD8 and Granzyme B staining, developed with Opal 570 and Opal 690. Fluorescent signals were acquired using the Vectra system or PhenoCycler-Fusion platform (Akoya Biosciences).

For multiplex immunohistochemistry (mIHC) analysis of mouse frozen tumor sections, the slides were firstly fixed with 4% paraformaldehyde (PFA), followed by permeabilization with 0.1% Triton X-100. Slides were then blocked with 2% bovine serum albumin (BSA) for 30 minutes at room temperature. Following blocking, sections were incubated overnight at 4 ^{o}C with a cocktail of fluorophore-conjugated primary antibodies including CD8-Alexa Fluor 555 (AF555), CD326 (EpCAM)-Alexa Fluor 594 (AF594), CD73-PerCP-CY5.5, H-2Kq (MHC I)-FITC, and IFN-γ-PECy7. Single-antibody stained slides were prepared in parallel and served as the essential reference controls for generating the spectral library required for accurate spectral unmixing in the InForm software (AKOYA). The fluorescent signals were acquired using the PhenoCycler-Fusion (Akoya Biosciences) platform.

Acquired images were analyzed using a trained algorithm within the InForm software (AKOYA). The algorithm first performed cell segmentation based on DAPI nuclear staining. It then executed cell phenotyping, classifying cells based on their specific marker expression patterns. The resulting quantitative data, including composite images, cell segmentation masks, and phenotyping results, were exported from InForm. This exported data was then subjected to further quantitative analysis of cellular densities and protein intensities using the R-based phenoptrReports and phenoptr software packages (AKOYA Biosciences). Spatial relationships between $CD73^{+}$/$CD73^{-}$ $PanCK^{+}$ tumor cells and $CD8^{+}$ T cells were quantified using the *Spatial Map Reviewer* tool in phenoptrReports (AKOYA). CD73, pAKT, and SMAD4 expression in $PanCK^{+}$ tumor cells were measured using InForm software. CD73 expression was dichotomized into high and low groups based on the median value.

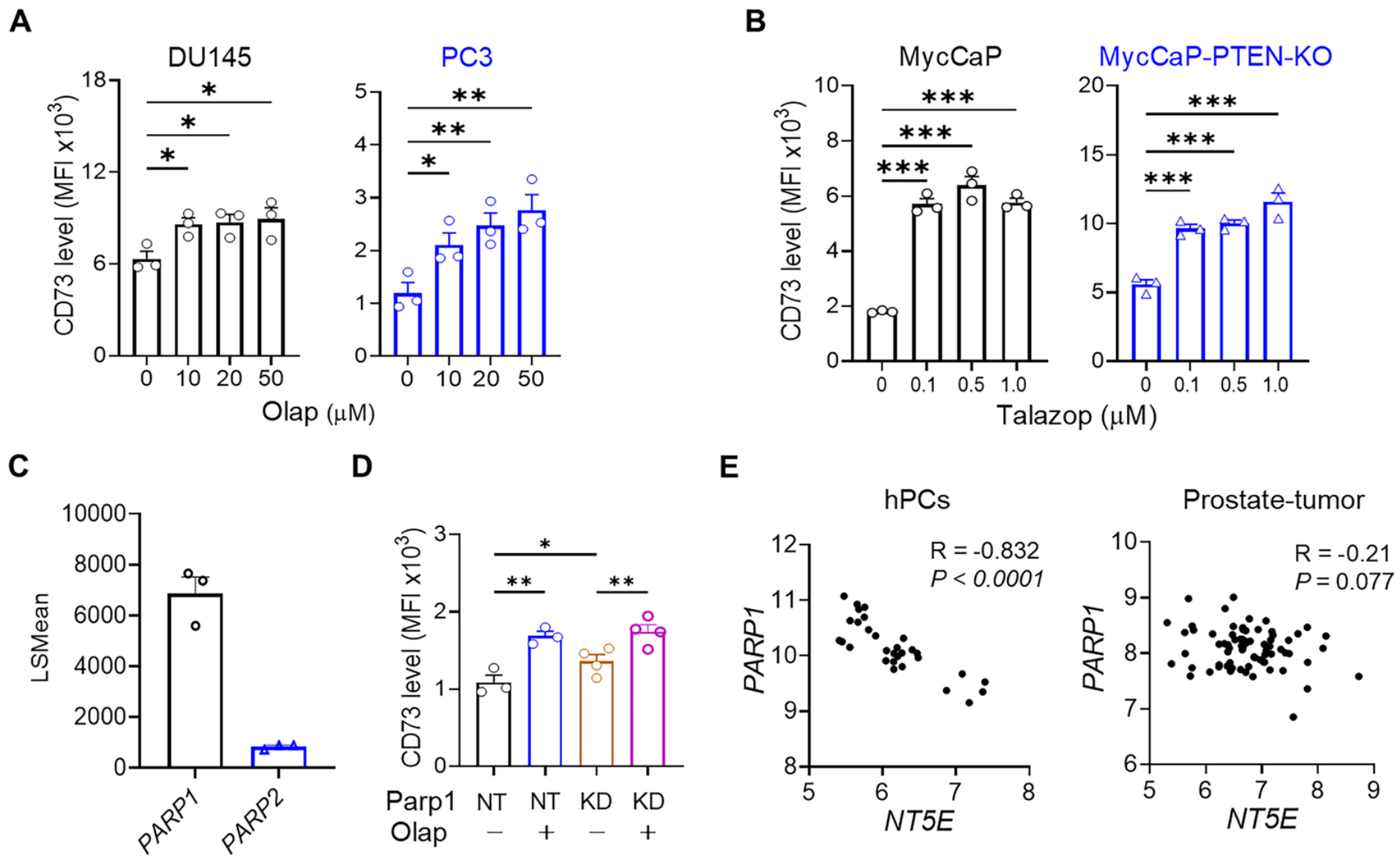

Supplementary Fig. 1. PARPi increases CD73 expression. (**A**) Flow cytometry analysis of CD73 expression in DU145 and PC3 human prostate cancer cells treated with Olaparib. (**B)** Flow cytometry analysis of CD73 levels in MycCaP and MycCaP-PTEN-KO cells following treatment with Talazoparib. ((**C**) Basal expression levels of *PARP1* and *PARP2* in MycCaP cells. (**D**) Flow cytometry analysis of surface CD73 levels in MycCaP cells with/without *PARP1* knockdown, in the presence or absence of 20 μM Olaparib treatment for 24 hours. (**E**) Correlation analysis of *PARP1* and *NT5E* gene expression in human prostate cancer cell lines (GSE50936, left) (n = 30) and tumor tissues (GSE2109, right) (n = 72). Results are representative of 3 (**A**, **B**, and **D**) independent experiments. Data were analyzed by one-way ANOVA with Tukey post-test correction (**A and B**), two-way ANOVA (**D**), or correlation analysis (**E**). Data plotted are mean ± SEM from biological replicates. *$P < 0.05$, ** $P < 0.01$, *** $P < 0.001$.

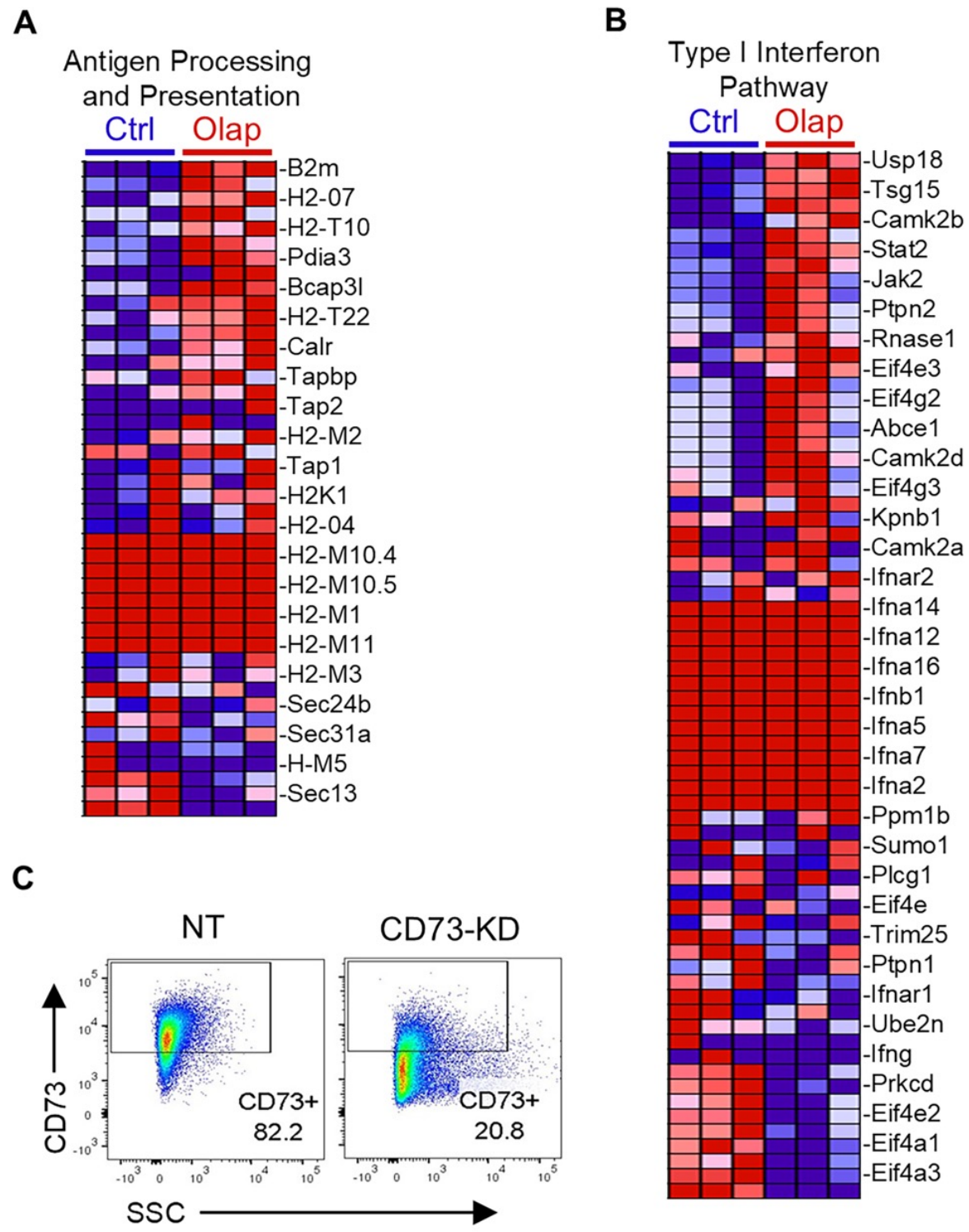

Supplementary Fig. 2. PARPi enhances tumor cell–intrinsic immunogenicity. (**A** & **B**) Heatmaps showing individual DEGs associated with the antigen processing and presentation pathway (**A**) and the type I interferon signaling pathway (**B**) in Olaparib-treated versus control cells. (**C**) Flow cytometry analysis of surface CD73 expression in MycCaP-NT (Non-target) and MycCaP-CD73-KD.

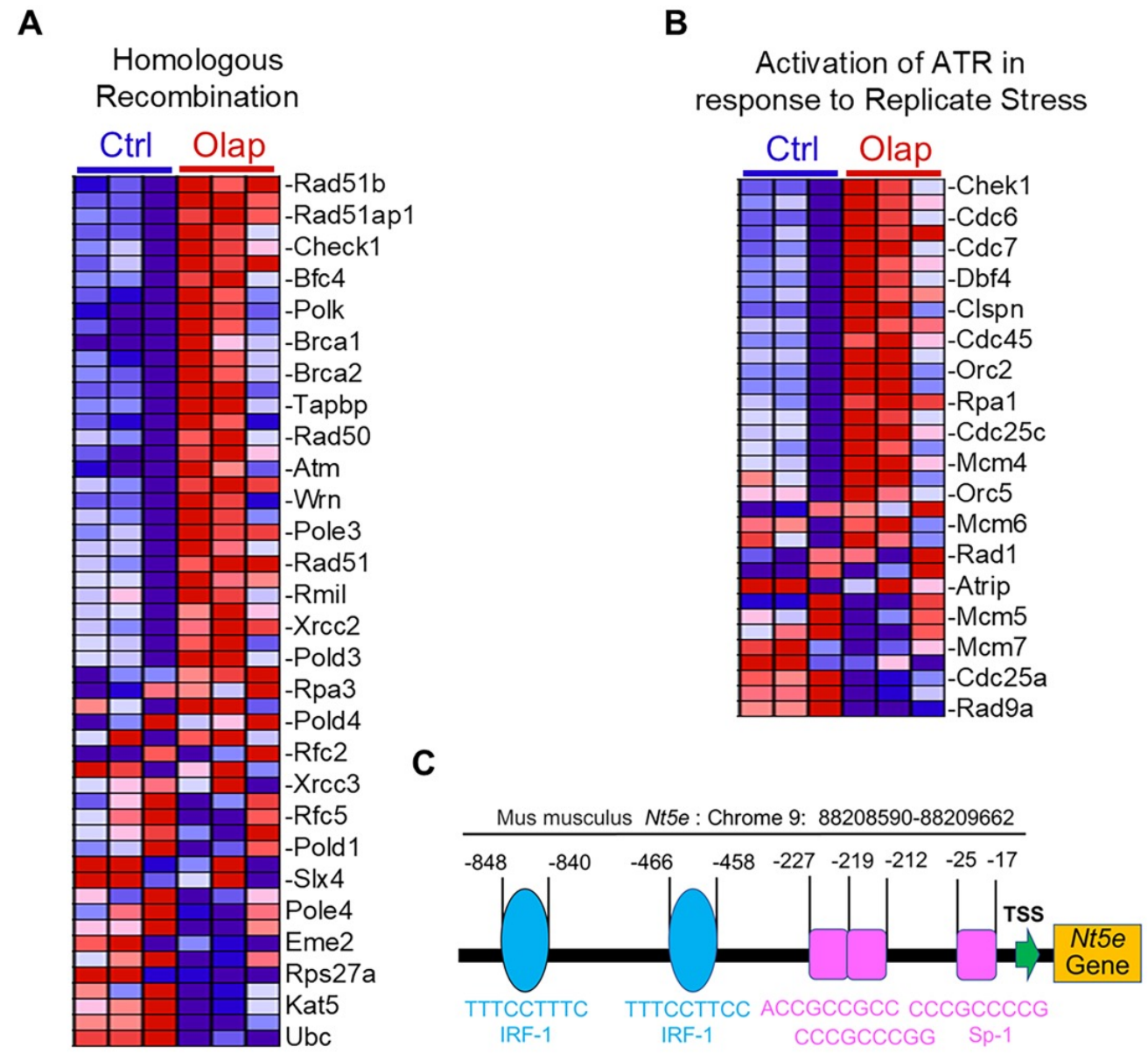

Supplementary Fig. 3. PARPi enhances DNA damage repair pathways. (**A** & **B**) Heatmaps showing DEGs associated with Homologous recombination (**A**) and Activation of ATR in response to replicate stress (**B**) in Olaparib-treated versus control cells. (**C**) Schematic prediction of potential IRF1 and SP1 transcription factor binding sites in the mouse *Nt5e* gene.

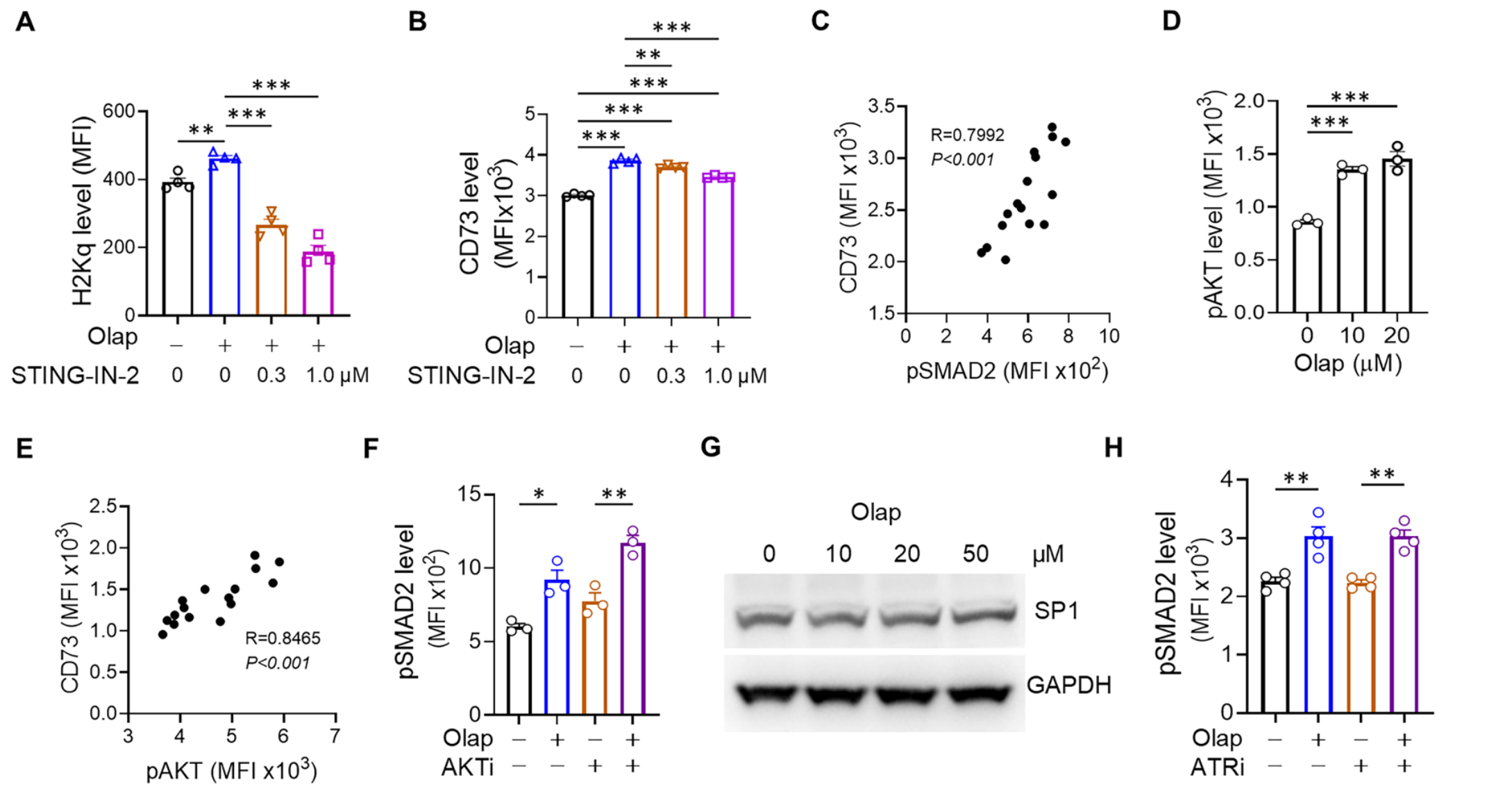

Supplementary Fig. 4. Regulation of CD73 expression by Olaparib. (**A & B**). Flow cytometric measurement of H2Kq (**A**) and CD73(**B**) levels in MycCaP cells pre-treated with STING-IN-2 at 0.3 and 1.0 µM followed by Olaparib treatment at 20 µM. (**C**) Flow cytometry analysis showing the association between CD73 expression levels and pSMAD2 intensity in MycCaP cells. (**D**) Flow cytometric assessment of pAKT intensity in MycCaP cells treated with Olaparib at 10 or 20 µM. (**E**) Flow cytometry analysis showing the association between CD73 expression levels and pAKT intensity in MycCaP cells. (**F**) Flow cytometric measurement of pSMAD2 levels in MycCaP cells treated with Olaparib, an AKT inhibitor, or both agents. (**G**) Western blot analysis of SP1 protein expression levels in MycCaP cells following Olaparib treatment. (**H**) Flow cytometric measurement of pSMAD2 levels in MycCaP cells treated with Olaparib, an ATR inhibitor, or both agents. Results are representative of 2 (**A**, **B, D** and **F**) or 3 (**C**, **E**, **G**, and **H)** independent experiments. Data were analyzed by one-way ANOVA with Tukey post-test correction (**D**), two-way ANOVA (**A**, **B**, **F**, and **H**), or correlation analysis (**C** and **E**). Data plotted are mean ± SEM from biological replicates. *$P < 0.05$, ** $P < 0.01$, *** $P < 0.001$.

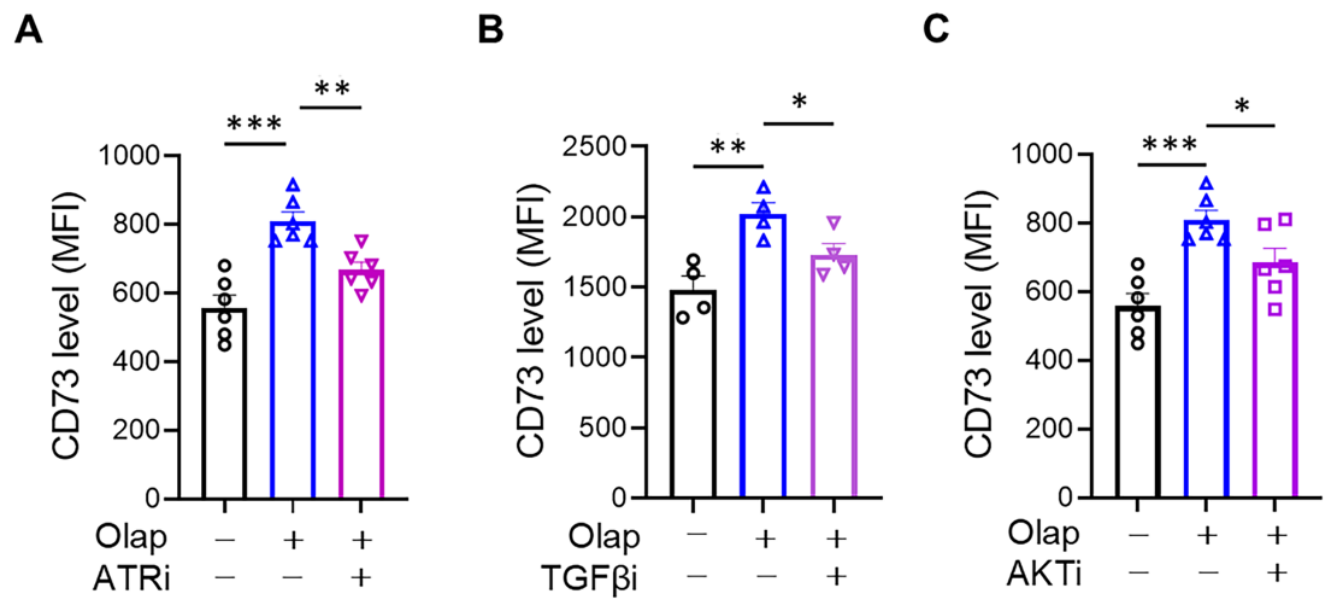

Supplementary Fig. 5. Olaparib enhances CD73 expression in human prostate cancer cells through the ATR and TGF-β signaling pathways. (**A-C**) Flow cytometric analysis of CD73 expression in DU145 cells pre-treated with an ATR inhibitor at 0.1 μM (**A**), a TGF-β inhibitor at 2 μM (**B**) and an AKT inhibitor at 0.1 μM (**C**), followed by 24 hours Olaparib treatment at 20 μM. Results are representative of two independent experiments. Data were analyzed by two-way ANOVA. Data plotted are mean ± SEM from biological replicates. *$P < 0.05$, ** $P < 0.01$, *** $P < 0.001$.

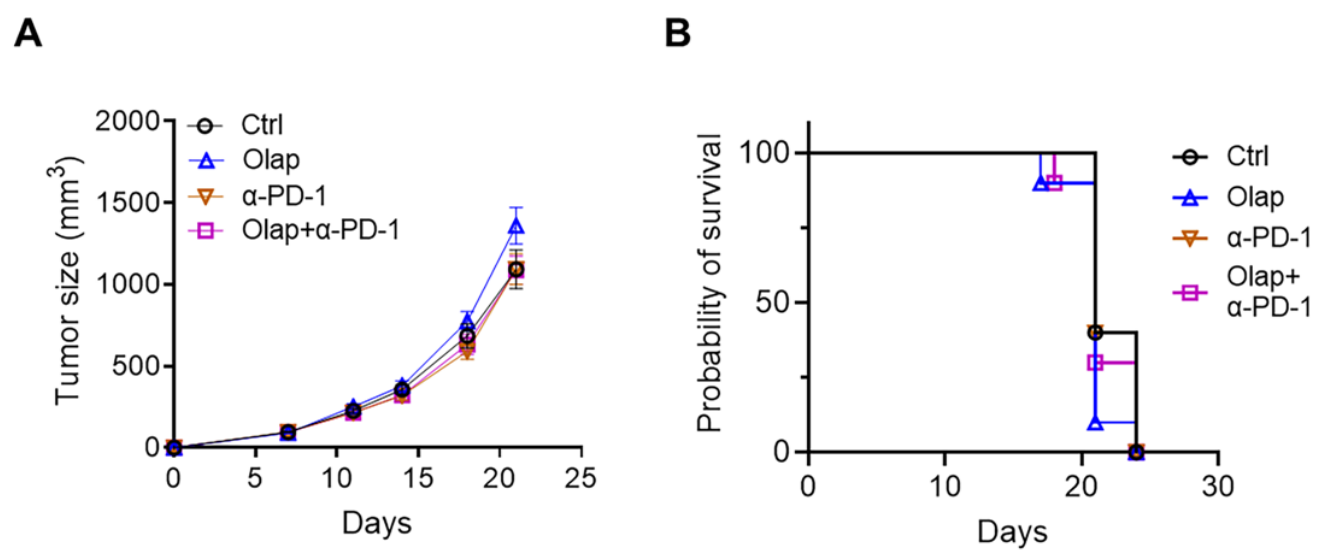

Supplementary Fig. 6. Ola combined with α-PD-1 fails to inhibit MycCaP tumor growth. (**A**) Tumor growth and (**B**) survival curves of MycCaP tumors treated with control (Ctrl), Olaparib alone, α-PD-1 alone, or the combination (n = 10). Data were analyzed by two-way ANOVA (**A**) or log-rank test for survival curves (**B**). Data plotted are mean ± SEM from biological replicates. Data were analyzed by two-way ANOVA (**B**-**I**, **K**, and **M**-**O**) or 2-tailed t test (**J** and **L**). Data plotted are mean ± SEM from biological replicates.

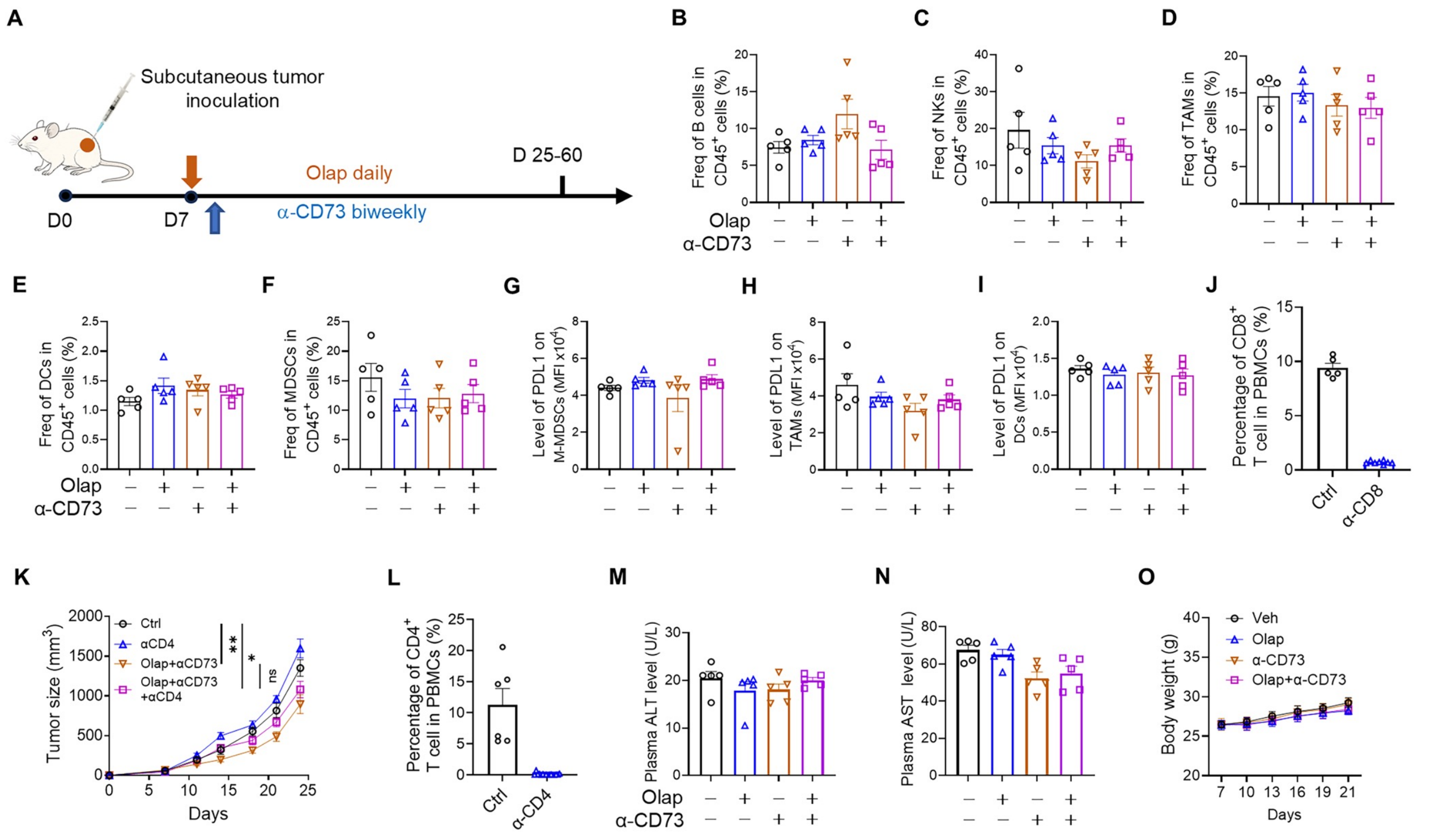

Supplementary Fig. 7. Olaparib and anti-CD73 (α-CD73) combination treatment schema and immune profiling of the tumor microenvironment. (**A**) Schematic diagram of the treatments with Olaparib (10 mg/kg, daily), α-CD73 (150 ug/mouse, biweekly) and combined Olaparib with α-CD73 after the MycCaP tumor cell inoculation (*n* = 5). Flow cytometry analysis of tumor-infiltrating immune populations, including B cells (**B**), NK cells (**C**), tumor-associated macrophages (TAMs) (**D**), dendritic cells (DCs) (**E**), and myeloid-derived suppressor cells (MDSCs) (**F**), across treatment groups. Flow cytometry analysis of PD-L1 expression on tumor-infiltrating monocytic MDSCs (M-MDSCs) (**G**), TAMs (**H**), and DCs (**I**) from treated mice. (**J**) Proportion of $CD8^+$ T cells in peripheral blood of MycCaP-bearing mice receiving combined Olaparib and α-CD73 with/without $CD8^+$ cell depletion (*n* = 8). (**K**) Tumor growth in MycCaP-bearing mice treated with/without combined Olaparib and α-CD73 following $CD4^+$ cell depletion (*n* = 7-8). ns, not significant. (**L**) Proportion of $CD4^+$ T cells in peripheral blood of MycCaP-bearing mice receiving combined Olaparib and α-CD73 with/without $CD4^+$ cell depletion (*n* = 6). (**N**) Plasma alanine amintransferase (ALT) level in MycCaP-bearing mice following the indicated treatments (*n* = 5). (**O**) Plasma aspartate aminotransferase (AST) level in MycCaP-bearing mice following the indicated treatments (*n* = 5). (**P**) Body weight changes were monitored every 3 days to assess potential treatment-related toxicity in treated MycCaP-bearing mice (*n* = 6). Data were analyzed by two-way ANOVA (**B**-**I**, **K**, and **M**-**O**) or 2-tailed student's *t* test (**J** and **L**). Data plotted are mean ± SEM from biological replicates. *$P < 0.05$, ** $P < 0.01$, *** $P < 0.001$, ns, non- significant

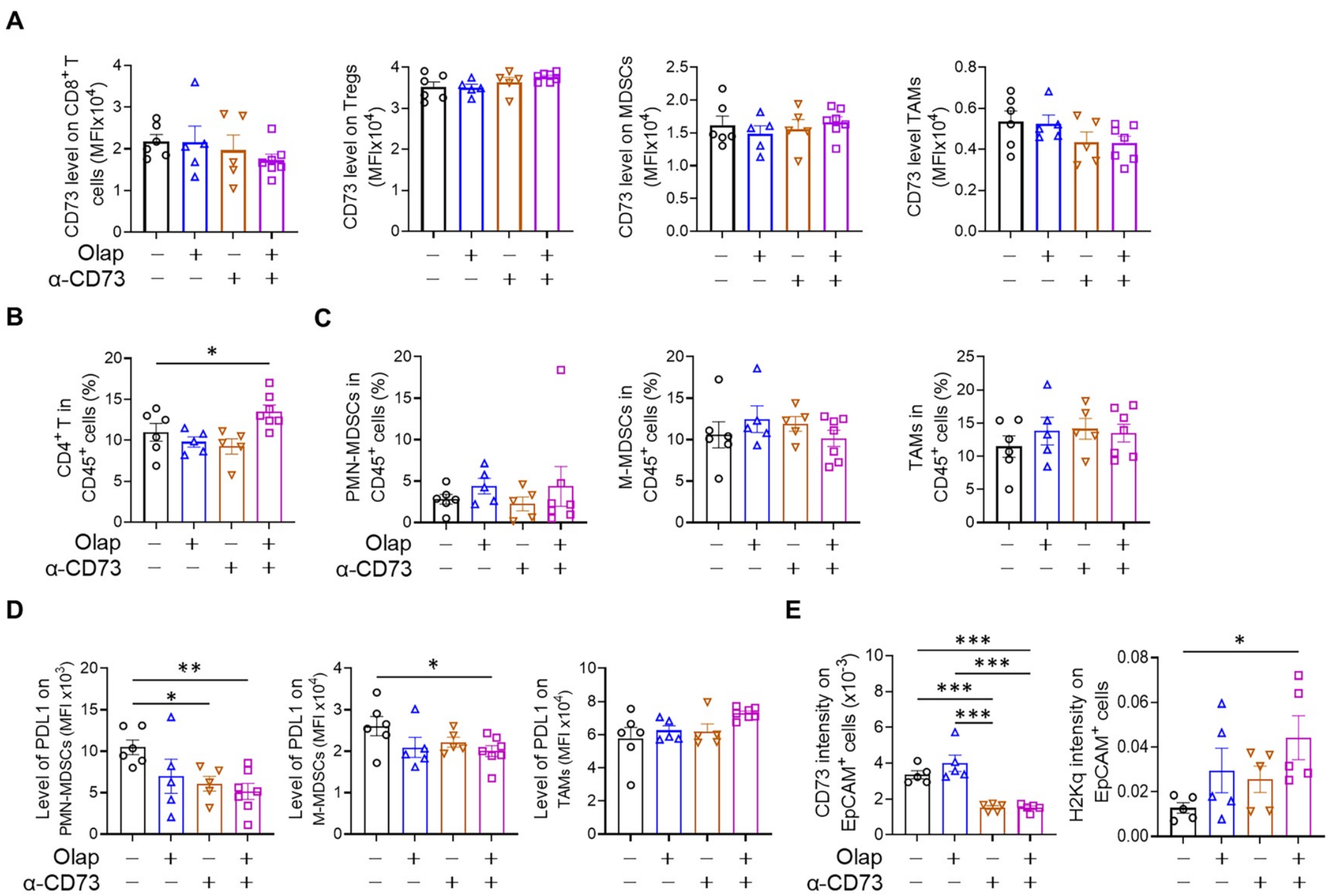

Supplementary Fig. 8. Olaparib plus anti-CD73 combination modulates the tumor immune microenvironment in orthotopic prostate tumors. (**A**) Flow cytometry analysis of expression levels of CD73 on $CD8^+$ T cells, Tregs, MDSCs and TAMs across indicated treatment groups (*n* = 5-7). (**B-D**) Flow cytometry analysis of the frequencies of $CD4^+$ (**B**), and PMN-DMSCs, M-MDSCs or TAMs (**C**) across treatment groups (*n* = 5-7). (**D**) PD-L1 expression on PMN-MDSCs, M-MDSCs and TAMs across treatment groups (*n* = 5-7). (**E**) mIHC analysis of CD73 and MHC-I (H2Kq) expression on EpCAM+ tumor cells across treated tissues (*n* = 5). Data were analyzed by two-way ANOVA. Data plotted are mean ± SEM from biological replicates. *$P < 0.05$, ** $P < 0.01$, *** $P < 0.001$.

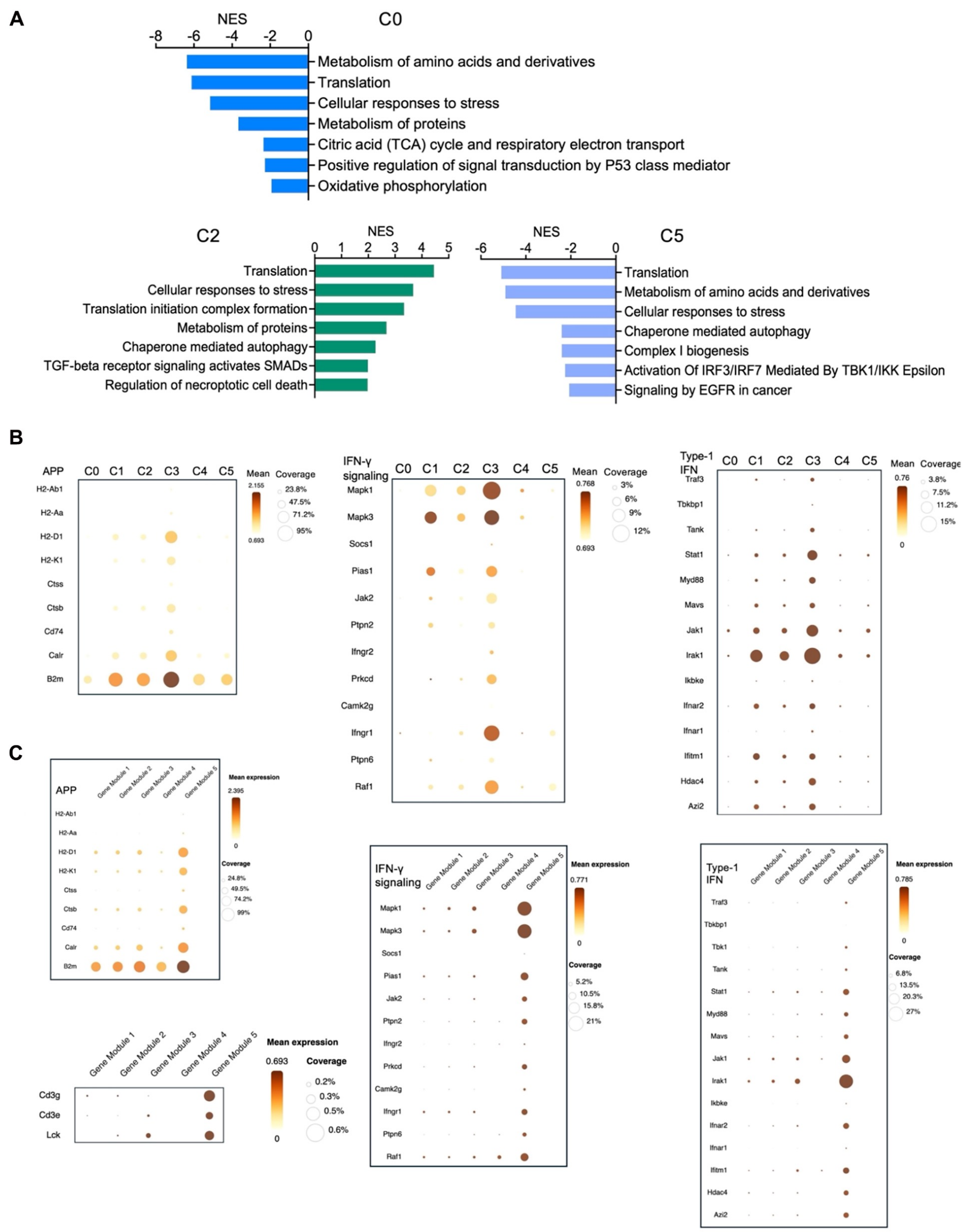

Supplementary Fig. 9. Spatial transcriptomics (ST) analysis reveals distinct immunocompetent regions associated with combination treatment. (**A**) Using SpatialX Software, the significantly upregulated and downregulated genes in each cluster were determined and subjected to GO enrichment analysis (Rectome) from the visium data sets . The top GO terms of each cluster presented as bar graph with normalized enrichment score (NES). (**B**) The expression of enriched gene sets for APP signature, IFN-γ signaling and Type-1 IFN response across Louvain clusters. (**C**) The expression of enriched gene sets for APP signature, IFN-γ signaling, Type-1 IFN response and T cell signature across individual gene modules defined by region segmentation analysis.

Table 1

Antibodies	Source	Catalog #
CD4-BV750 (GK1.5)	Biolegend	100467
CD8a-Spark Blue550 (53-6.7)	Biolegend	100780
CD45-BV570 (30-F11)	Biolegend	103135
CD3e-Percp5.5 (145-2C11)	Biolegend	100328
CD11b-BB515 (M1/70)	BD Horizon	564454
CD11c-AF532 (N418)	eBiosciences	58011482
CD19-BV785 (6D5)	Biolegend	115543
CD25-BV650 (PC61)	Biolegend	102038
CD44-PECy5 (IM7)	Biolegend	103010
CD69-BV510 (H1.2F3)	Biolegend	104532
CD73-APC (TY/11.8)	Biolegend	127210
IFN-γ-PECy7 (XMG1.2)	Biolegend	505826
TNF-α-BV650 (MP6-XT22)	Biolegend	506333
F4/80-PECy5 (BM8)	Biolegend	123114
MHCII-PE/Dazzle™ 594 (M5/114.15.2)	Biolegend	121606
CD163-PE (S15049F)	Biolegend	156704
CD274-BV605 (10F.9G2)	Biolegend	124321
CD279-APC-R700 (J43)	BD Biosciences	565815
Ly-6C-BV711 (HK1.4)	Biolegend	128037
Ly-6G/C-AF594 (RB6-8C5)	Biolegend	108448
Tim3-APC (B8.2C12)	Biolegend	134008
CD335(NKp46)-PerCp-eFluor710	eBiosciences	46-3351-82

CX3CR1-APC/Fire750 (SA011F11)	Biolegend	149039
CD86-FITC (GL-1)	Biolegend	105006
FoxP3-BV421 (MF-14)	Biolegend	126419
Ly108-BB700 (13G3)	BD Biosciences	742272
Ki67-PacificBlue (16A8)	Biolegend	652422
Tox-eFluor660 (TXRX10)	eBiosciences	50650282
CD103-BV480 (M290)	BD Biosciences	566118
KLRG1-PECy7 (2F1/KLRG1)	Biolegend	138416
CD45-APC-Cy7 (30-F11)	Biolegend	103116
CD8-BUV737 (53-6.7)	BD Biosciences	612759
CD4-FITC (GK1.5)	Biolegend	100406
CD279 (PD1)-PE (29F.1A12)	Biolegend	135206
CD8-AF555 (EPR21769)	abcam	Ab280863
CD326-AF594 (G8.8)	Biolegend	118222
CD73-PerCP-Cy5.5 (TY/11.8)	Biolegend	127214
H-2Kq-FITC (KH114)	Biolegend	115104
Zombie yellow	Biolegend	423103
Live/Dead fixable blue	ThermoFisher	L23105

Table. 2

	Antibodies				Fluorophore
Panel	Clones	Dilution	Venders	Catalog #	
	CD73 (D7F9A)	1:100	Cell signaling	13160s	Opal 650
	CD8 (C8/144B)	1:200	Cell signaling	70306s	Opal 540
	SMAD4 (D3R4N)	1:20	Cell signaling	46535s	Opal 520
	pAKT (D9E)	1:10	Cell signaling	4060s	Opal 570
	PanCK (C-11)	1:200	abcam	ab7753	Opal 690
