# Supplementary material for: CD73 inhibition overcomes adaptive immune resistance to PARP inhibition in models of prostate cancer
Source: J Clin Invest. 2026 Aug 18;136(19):e203785. doi: 10.1172/JCI203785 (PMC13626842; doi:10.1172/JCI203785)
Supplement: Supplementary file 2 [file jci-136-203785-s257.pdf]

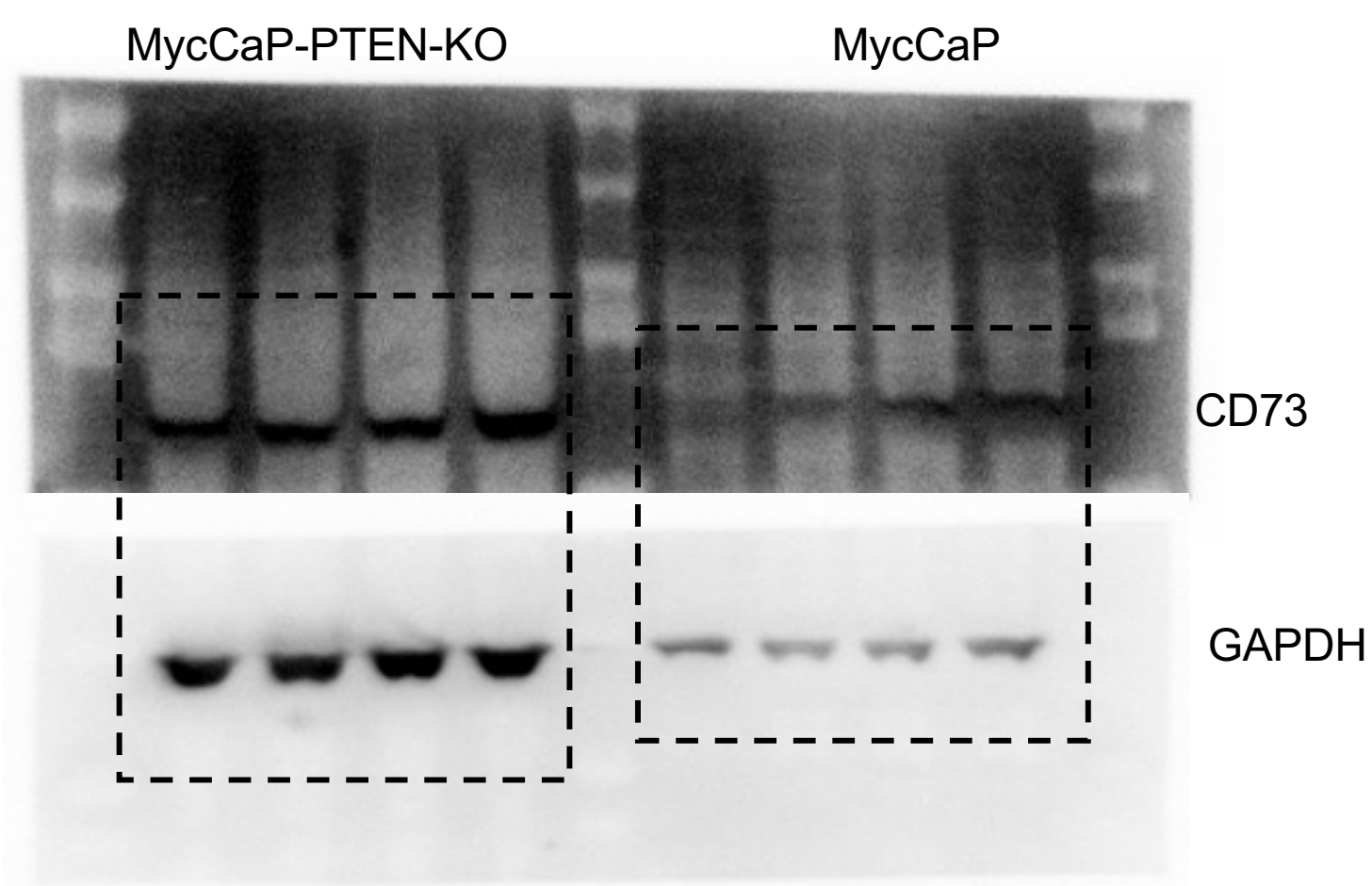

Figure 1. (E) Western blot analysis of CD73 protein expression

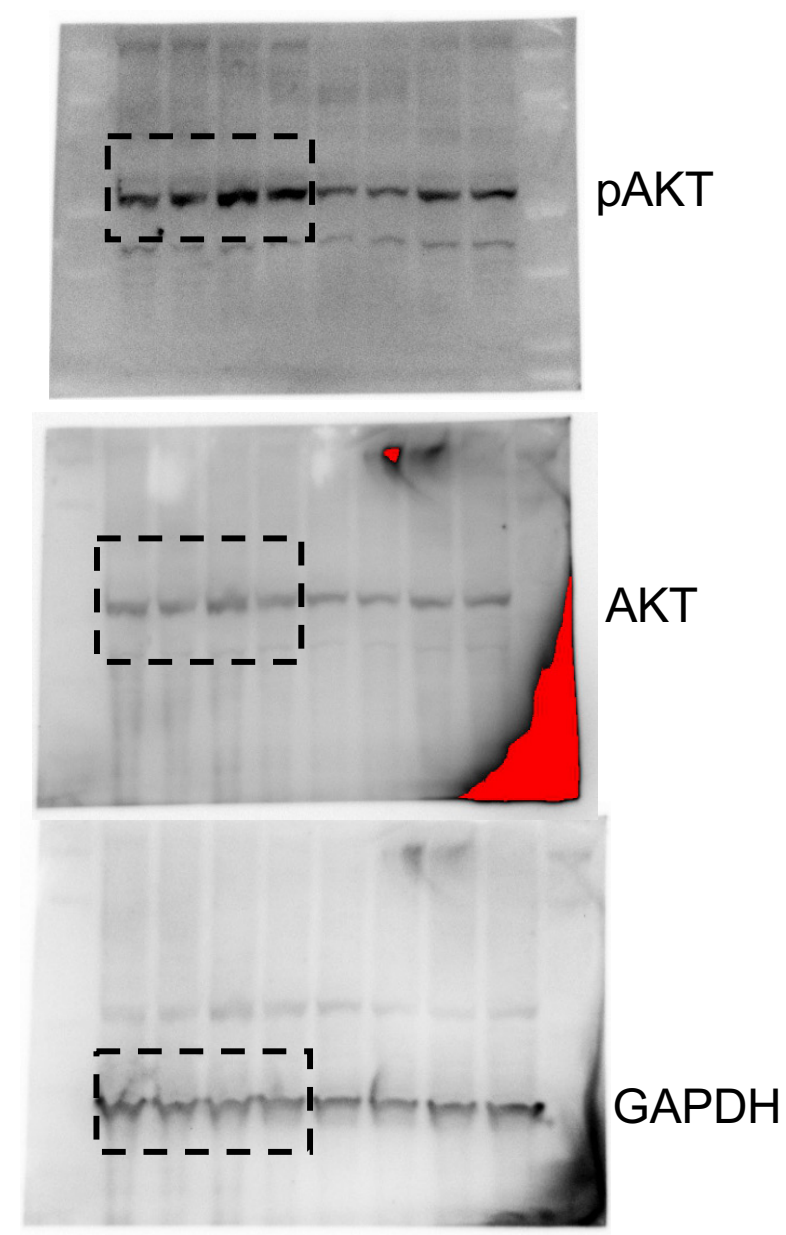

Figure 3. (E) Western blot analysis of pAKT protein expression

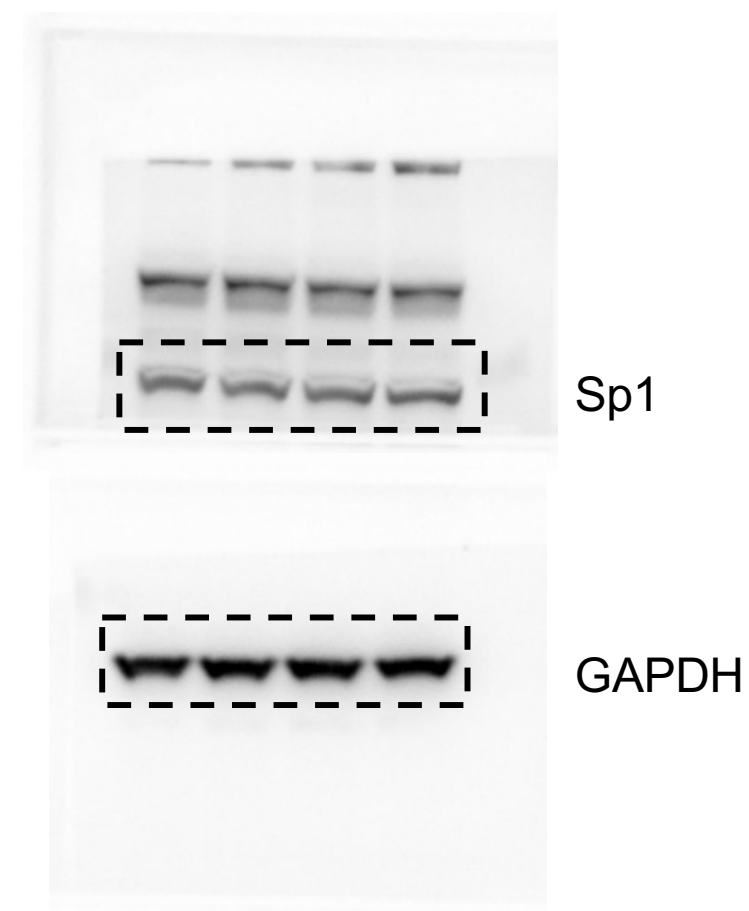

Supplementary Figure 4. (G) Western blot analysis of SP1 protein expression
